# Large-Scale Phosphoproteomic Study of *Arabidopsis* Membrane Proteins Reveals Early Signaling Events in Response to Cold

**DOI:** 10.3390/ijms21228631

**Published:** 2020-11-16

**Authors:** Md Mostafa Kamal, Shinnosuke Ishikawa, Fuminori Takahashi, Ko Suzuki, Masaharu Kamo, Taishi Umezawa, Kazuo Shinozaki, Yukio Kawamura, Matsuo Uemura

**Affiliations:** 1 United Graduate School of Agricultural Sciences, Iwate University, Morioka 020-8550, Japan; mostafa.kamal42@gmail.com (M.M.K.); ykawa@iwate-u.ac.jp (Y.K.); 2 Graduate School of Bio-Applications and Systems Engineering, Tokyo University of Agriculture and Technology, Koganei 184-8588, Japan; shinnosuke-ishikawa@keio.jp (S.I.); taishi@cc.tuat.ac.jp (T.U.); 3 Gene Discovery Research Group, RIKEN Center for Sustainable Resource Science, 3-1-1 Koyadai, Tsukuba 305-0074, Japan; fuminori.takahashi@riken.jp (F.T.); kazuo.shinozaki@riken.jp (K.S.); 4 Department of Biochemistry, Iwate Medical University, Yahaba 028-3694, Japan; kosuzu@iwate-med.ac.jp (K.S.); mkamo@iwate-med.ac.jp (M.K.); 5 Department of Plant-Bioscience, Faculty of Agriculture, Iwate University, Morioka 020-8550, Japan

**Keywords:** *Arabidopsis*, cold response, membrane phosphoproteomics, stress perception, stress signaling, cold stress

## Abstract

Cold stress is one of the major factors limiting global crop production. For survival at low temperatures, plants need to sense temperature changes in the surrounding environment. How plants sense and respond to the earliest drop in temperature is still not clearly understood. The plasma membrane and its adjacent extracellular and cytoplasmic sites are the first checkpoints for sensing temperature changes and the subsequent events, such as signal generation and solute transport. To understand how plants respond to early cold exposure, we used a mass spectrometry-based phosphoproteomic method to study the temporal changes in protein phosphorylation events in *Arabidopsis* membranes during 5 to 60 min of cold exposure. The results revealed that brief cold exposures led to rapid phosphorylation changes in the proteins involved in cellular ion homeostasis, solute and protein transport, cytoskeleton organization, vesical trafficking, protein modification, and signal transduction processes. The phosphorylation motif and kinase–substrate network analysis also revealed that multiple protein kinases, including RLKs, MAPKs, CDPKs, and their substrates, could be involved in early cold signaling. Taken together, our results provide a first look at the cold-responsive phosphoproteome changes of *Arabidopsis* membrane proteins that can be a significant resource to understand how plants respond to an early temperature drop.

## 1. Introduction

Throughout the Earth’s history, plants have evolved adaptive mechanisms against multiple abiotic and biotic stresses. Cold stress, which results from both chilling (0–10/15 °C) and freezing (<0 °C) conditions, is one of the major abiotic stresses that happens all over the world and affects every process in the plant life cycle, including growth, development, survival, and productivity [1]. Cold-stress responses vary with plant species. Agronomically important plants, such as rice (*Oryza sativa*), corn (*Zea mays*), banana (*Musa* spp.), and tomato (*Lycopersicon esculentum*), originated from tropical and subtropical regions and are sensitive to cold stress. [2,3,4,5]. On the other hand, plants native to temperate climatic zones can take advantage of the colder autumn temperature and prepare themselves for the upcoming freezing conditions using an adaptive strategy called cold acclimation [4,5,6]. Therefore, perception of and response to a drop in temperature is essential for plants that originated in tropic and sub-tropic regions to deploy survival mechanisms and for those in temperate climatic zones to start the acclimation processes and prepare for upcoming freezing stress. Responses to cold under acclimating and freezing conditions are studied extensively on both genomic and proteomic levels [7,8,9,10,11,12,13]. However, there is little or no information available on how plants sense temperature drops over a short time (i.e., seconds and minutes) and how they respond and form adaptive signals to the cold. Sensing early changes is an essential step for the plant to initiate the adaptive process and survive under cold temperatures. Furthermore, how plants respond to early temperature drops is necessary to develop cold-resistant/-tolerant plant varieties and secure crop supply in the global climate change era.

The cell wall (CW), the extracellular/apoplastic region, and the plasma membrane (PM) are the outermost layers of a plant cell. Therefore, upon exposure to external environmental stimuli (biotic and abiotic), these layers are the most likely to receive, generate, and transmit signals further downstream and ultimately to the nuclei to form an appropriate response [14,15,16,17]. PM is the outermost living part of the cell and has received the most attention for cold response studies in plants [7,8,10,11,13,18,19]. Few studies have also emerged on CW modifications during prolonged cold acclimation and in freezing response [18,20,21]. These studies indicate that the outer layers work in concert, as the first checkpoint to sense and generate signals from cold and other environmental factors, by changing their physicochemical conformation and composition and utilizing the power of posttranslational modifications (PTMs), such as phosphorylation of receptor-like kinases (RLKs) and other membrane-localized kinases [19,22,23,24,25]. Within 30 min to cold exposure at 4 °C, the PM rigidifies and increases Ca^2+^ influx into the cell, which in turn activates Ca^2+^/calmodulin (CaM)-regulated receptor-like kinase1 (CRLK1), leading to mitogen-activated protein kinase (MAPK) pathway activation via a chain of phosphorylation events [23,26]. The MAPK pathway is one of the canonical signaling pathways that enhance freezing tolerance in *Arabidopsis* [25,27]. A phosphoproteomic study of cold-tolerant and cold-sensitive bananas (*Musa* spp.) showed that Thr31 phosphorylation of MAPK kinase 2 (MKK2) is an essential component of the cold tolerance mechanism [28]. CRLK1, MEKK1 (MAPK kinase kinase 1), and MKK2 are all reported to be localized in the PM [29,30]. Similarly, another membrane-localized kinase, named cold-responsive protein kinase 1 (CPPK1), was activated upon prolonged cold exposure and directly sent a signal to the nucleus to fine-tune the C-repeated binding factor (CBF) signaling pathway by phosphorylating its 14-3-3 protein substrate [31]. Another membrane-bound receptor-like kinase, receptor-like protein kinase 1 (RPK1), also increase cold tolerance along with tolerance to other abiotic stresses [32]. CW, apoplast, PM-bound and localized RLKs, and transmembrane kinases (TMKs), and their signaling events, are better documented in biotic stresses than abiotic stresses [21,33]. Therefore, further studies are needed to discover cold-stress-responsive RLKs and other protein kinases that are localized in the membranes and membrane-adjacent extracellular spaces.

Furthermore, these examples strongly indicate that protein phosphorylation of membrane-bound kinases and their targets (e.g., transporters, ion channels, and transcription factors) plays a crucial role in cold and other abiotic stress-triggered molecular events. Along with stress signal transduction, protein phosphorylation/dephosphorylation is also involved in regulating diverse biological and molecular functions, such as cell division, differentiation, gene expression, hormone perception, homeostasis, and metabolism. Therefore, protein phosphorylation is one of the most important PTMs in plants [34,35,36,37]. Signals for all the processes mentioned above originate at the PM and its adjacent regions either by a change in its physicochemical properties or by phosphorylation of the kinases associated with these regions. Therefore, studying and profiling the membrane-localized phosphoproteome and kinome on a large scale at earlier stages of cold exposure will help us to understand the plant’s response to cold and other signaling events related to cold response.

Mass spectrometry (MS)-based shotgun phosphoproteomics has emerged as a powerful analytical tool to study protein phosphorylation on a large scale. MS-based phosphoproteomics has become a standard method to study global changes in phosphorylation dynamics [17,38,39,40,41,42]. Although significant technical improvements have been made to study the global or total phosphoproteins, characterization of membrane phosphoproteins is still challenging. A low abundance, short-lived dynamic signaling events under specific conditions, contamination from other non-membrane proteins, and the overabundance of non-phosphopeptides are a few of the major roadblocks in studying membrane phosphoproteins [36,43,44]. Here, to improve the detection of low-abundance membrane phosphopeptides, we used a well-established membrane protein extraction method [45] with minor modifications and enriched phosphopeptides with TiO_2_. We further analyzed the TiO_2_-enriched membrane phosphopeptides using a high-resolution mass spectrometer coupled with a long monolithic column to improve the separation capacity. We prepared the microsomal membrane fractions (MMFs) from two-week-old *Arabidopsis* aerial parts after exposure to 2 °C for short periods (i.e., 5, 15, 30, or 60 min). Using these samples, we successfully identified the phosphorylated proteins from different membrane-localized kinases and their substrates and analyzed their dynamics upon short cold exposure.

## 2. Results

### 2.1. Description of the Phosphoproteomic Data of Early Cold Treatment in Arabidopsis

**Figure 1:**
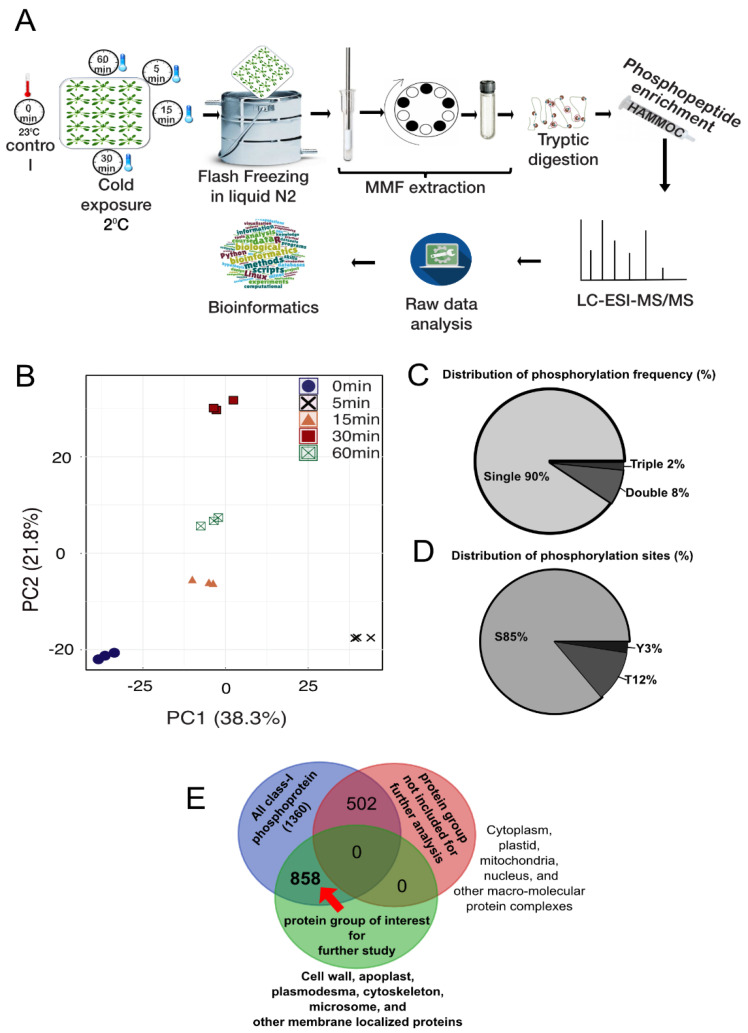
Analysis of the cold-responsive phosphoproteins in *Arabidopsis thaliana*. (**A**) Experimental design and analytical workflow. (**B**–**E**) Quantitative analysis of the phosphopeptides: (**B**) Principal component analysis (PCA) of all the identified peptides (including phosphorylated and non-phosphorylated peptides). The times indicate the cold exposure duration: 5 to 60 min. (**C**) Frequency distribution of all the identified phosphopeptides based on the number of phosphorylation sites and phosphorylated amino acids. (**D**) Frequency of phosphosites per peptide: S, serine; T, threonine; Y, tyrosine. (**E**) Gene-ontology enrichment of cellular localization and the distribution of the proteins of interest. Class-I phosphoprotein: phosphopeptides with a mascot variable modification confidence score ≥ 0.75. The red arrow indicates the number of proteins comes from the MMFs.

The purpose of this study is to elucidate the signaling events in response to cold stress in the model plant *Arabidopsis* by a time-course (0, 5, 15, 30, and 60 min)-based phosphoproteomic study (Figure 1A). We identified a total of 2795 unique phosphopeptides, which were phosphorylated in at least two biological replicates at all five time points, and these phosphopeptides constitute a total of 1360 proteins (Table S1A,B). We performed a principal component analysis (PCA) to evaluate the quality of the biological replicates at each time point. PCA analysis showed all biological replicates at each time point cluster together, and simultaneously, were separated from each other (Figure 1B). Furthermore, about 90% of these phosphopeptides were singly phosphorylated (Figure 1C). From the identified phosphopeptides, a total of 2930 phosphosites were enriched, where the most frequent phosphorylation was observed in serine (S, 85%), followed by in threonine (T, 12%) and tyrosine (Y, 3%) (Figure 1D). The frequency distribution of the phosphorylation sites was similar to those previously reported in plant phosphoproteomic studies [44,45,46,47,48,49,50]. 

The MMF used in the present study is a mixture of various cellular membranes and compartments and enriched in lighter membranes such as the PM, endoplasmic reticulum, Golgi body, and vacuole, but it also contains fractions from the extracellular space, cell wall, apoplast, cytoskeleton, plasmodesmata, cytosol, mitochondria, plastid, nucleus, and other cellular compartments. Because the objective of our study was to observe the phosphorylation-level changes in proteins potentially involved in the early cold response in the MMF, we performed an over-representation analysis on the cellular localization of the proteins and divided them into two groups based on significant enrichment (Fisher test, *p* < 0.05): (A) the protein group of interest, which includes proteins from the PM, endoplasmic reticulum, Golgi body, vacuole, extracellular space, cell wall, apoplast, cytoskeleton, and plasmodesmata; and (B) the sub-portion of proteins from the cytosol, mitochondria, plastid, nucleus, and macromolecular complexes. Out of 1360 phosphoproteins, 858 were from Group A. Our detailed study in the following sections only included group A proteins, and the proteins from Group B were not included in further analyses (Figure 1E). Group A contains 1821 phosphopeptides and 1894 phosphosites. Next, we analyzed the relative changes in the phosphopeptides after cold exposure compared to the control (0 min cold exposure) in a time-course manner (Table S1B). 

### 2.2. Temporal Profile of the Phosphopeptides in Response to Cold

Previous phosphoproteomic studies of osmotic stress and systemin treatment reported that the total protein abundance does not change within a time frame of minutes; instead, the peptide abundance comes from the changes in phosphorylation dynamics [42,48,51]. To determine whether this was the case under our experimental conditions, we performed a proteomic study on plants exposed to cold for up to 60 min. We did not see any significant changes in protein abundance (Table S2). Therefore, the changes in peptide abundance we observed in this study were due to changes in phosphorylation itself rather than de novo protein synthesis. 

**Figure 2:**
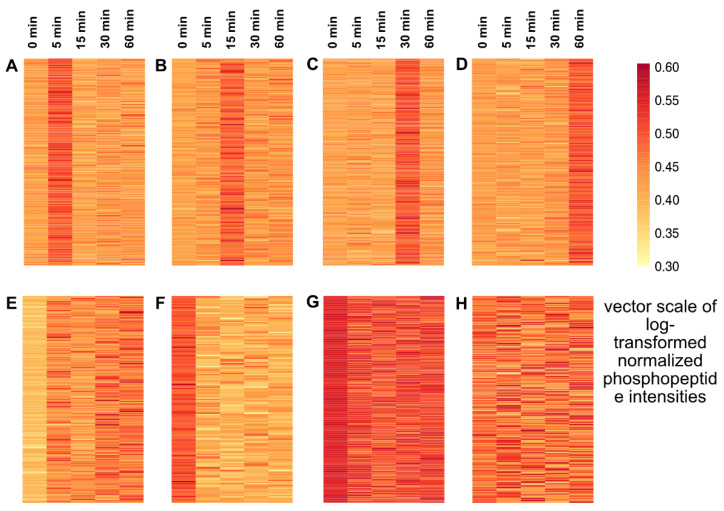
K-means clusters in the time-course analysis of phosphopeptide intensities in response to cold. Clusters are positioned based on their similar time-based characteristics. (**A**) Phosphorylation peaked at 5 min. (**B**) Phosphorylation peaked at 15 min. (**C**) Phosphorylation peaked at 30 min. (**D**) Phosphorylation peaked at 60 min. (**E**) Continuous phosphorylation under cold exposure. (**F**) Continuous dephosphorylation in response to cold. (**G**) Phosphopeptides dephosphorylate under cold exposure, but not continuously. (**H**) Unresponsive to cold exposure. The scale represents the vector transformation of the log2-transformed normalized phosphopeptide intensities.

We next studied the phosphorylation dynamics upon cold exposure using K-means clustering of the log-transformed normalized phosphopeptides from the Group A proteins. The temporal dynamics of phosphopeptides created eight characteristic K-means clusters (Figure 2). Upon cold exposure, a large number (481) of peptides were dephosphorylated (Figure 2, Clusters F and G). Among the dephosphorylated peptides, 140 peptides stayed in the dephosphorylated state relative to the control throughout the cold exposure period (Figure 2, Cluster F). Cluster G, which contains 341 peptides, showed a fluctuation in its dephosphorylation pattern, but none of these peptides showed increased phosphorylation relative to the control. Next, four characteristic clusters were found where peptide phosphorylation transiently peaked at each of the four cold exposure time points (Figure 2, Clusters A, B, C, D, and E). Cluster A peaked very early at 5 min with 243 phosphopeptides, and Cluster B with a peak at 15 min contained 177 phosphopeptides. We then grouped these two clusters as “early response” to cold exposure. The two clusters C (285 phosphopeptides) and D (209 phosphopeptides) peaked at later time points, 30 and 60 min, respectively. In Cluster E, peptides were immediately phosphorylated upon cold exposure and maintained phosphorylation states throughout the cold exposure period. Even though the peptides in this group (Cluster E) showed continuous phosphorylation, their peak intensities fluctuated at different time points. In further steps, we redistributed 177 phosphopeptides from Cluster E to Clusters A and B (early response) and Clusters C and D (late response) based on their highest peak intensities at each time point during cold exposure. Cluster H, containing 230 phosphopeptides, did not show any changes upon cold treatment compared to the control; it was classified as “unresponsive” and was excluded from further analysis. For quantitative analysis, we compared significant (Student’s *t*-test, *p*-value < 0.05) fold changes (cold exposed/non-exposed: 0.5 ≤ log_2_FC ≥ 1) for individual phosphorylated/dephosphorylated peptides in each cluster and visualized them in a heatmap (Figure S1). All the significantly (*p*-value < 0.05) phosphorylated peptides (1009) in the early and late response groups were subjected to gene ontology over-representation analysis (ORA) of molecular function (GOMF) and biological processes (GOBP), and motif analysis. 

### 2.3. Cold Signal Responsive Molecular Functions in Arabidopsis: GOMF

**Figure 3:**
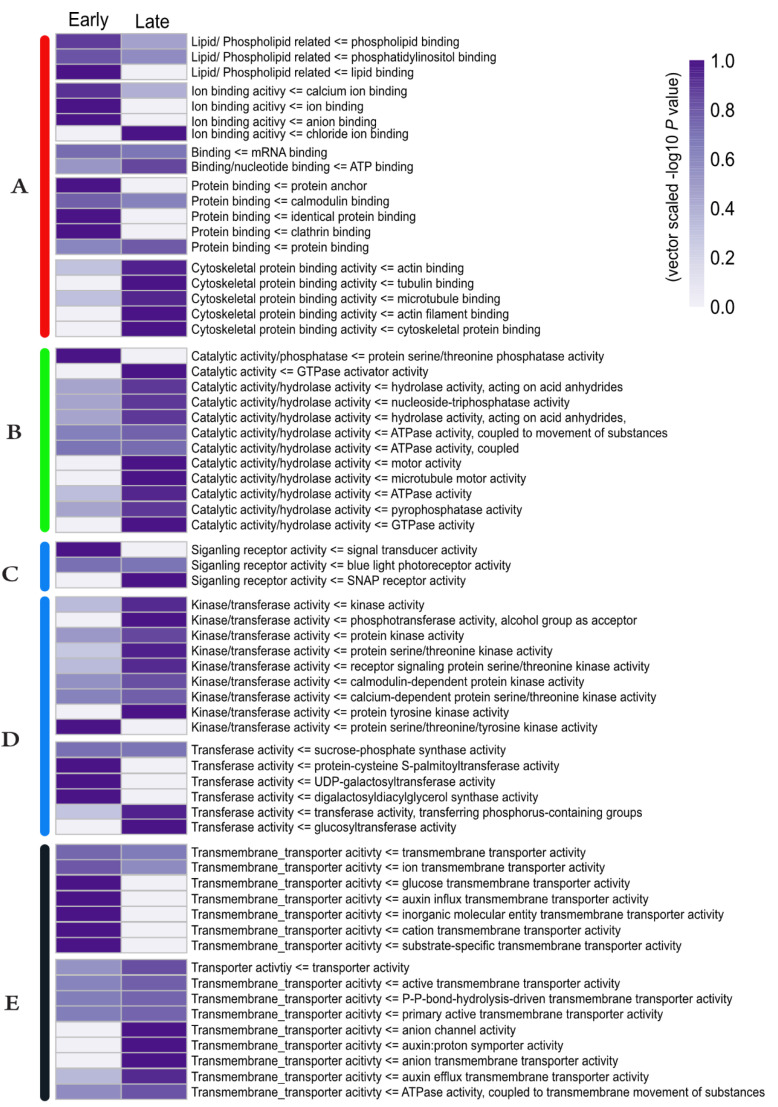
Gene ontology overrepresentation of molecular function (GOMF) for the phosphorylated proteins under cold exposure. Negative log 10 *p*-values of the GO terms enriched in the K-means clusters mentioned in Figure 2 were vector-scaled and visualized. Sub-clusters (**A**–**E**) are based on the similarity of GO terms extracted from ReViGO. Early, GO terms enriched within 5 to 15 min of cold exposure; Late, GO terms enriched within 30 to 60 min of cold exposure.

To gain insight into the biological relevance of phosphorylation, we performed an over-representation analysis (Fisher exact test) in the early response and late response group proteins. The overrepresented GO terms revealed characteristic shifts in the molecular function (GOMF) of these phosphoproteins in response to cold exposure. Proteins involved in binding, catalytic activity, signaling, and transport were phosphorylated upon cold exposure (Figure 3). Binding proteins in the early response group were mostly phospholipid, calmodulin, clathrin, calcium, and other ion-binding phosphoproteins. Interestingly, ATP, chloride ions, the cytoskeleton, and microtubule-binding phosphoproteins were highly abundant in the late response clusters, and nucleic acid binding phosphoproteins were over-represented in both clusters (Figure 3A). Phosphoproteins with catalytic/hydrolase activity were significantly over-represented in the late response clusters, except for phosphoproteins related to serine/threonine phosphatase activity (Figure 3B). Proteins related to blue light photoreceptor activity were in the phosphorylated state throughout the cold exposure period. Signal-transducing phosphoproteins were enriched only within 15 min of cold exposure, and soluble N-ethylmaleimide-sensitive factor (NSF) adaptor protein (SNAP) receptors were only phosphorylated in the late response group (Figure 3C). Signal transduction related proteins (kinase/transferase/transducers) were also significantly overrepresented after 30 min of cold stress; however, serine/threonine/tyrosine kinase, palmitoyltransferase, and galactosyltransferase activity-related phosphoproteins were only found in the early response group (Figure 3D). Transport and transmembrane transport-related phosphoproteins were enriched in the early and late response clusters, where transmembrane transporters involved in auxin influx and ion/cation/carbohydrate transportation were only phosphorylated within 15 min of cold exposure (Figure 3D). Transporters and transmembrane transporters involved in auxin efflux, auxin proton symporter, anion channel activity, and ATPase-mediated transportation were highly enriched in the late cold response clusters (Figure 3E). In brief, these results show a large number of proteins involved in important molecular functions are temporally phosphorylated under cold exposure. 

### 2.4. Cold Signal Responsive Biological Processes in Arabidopsis: GOBP

**Figure 4:**
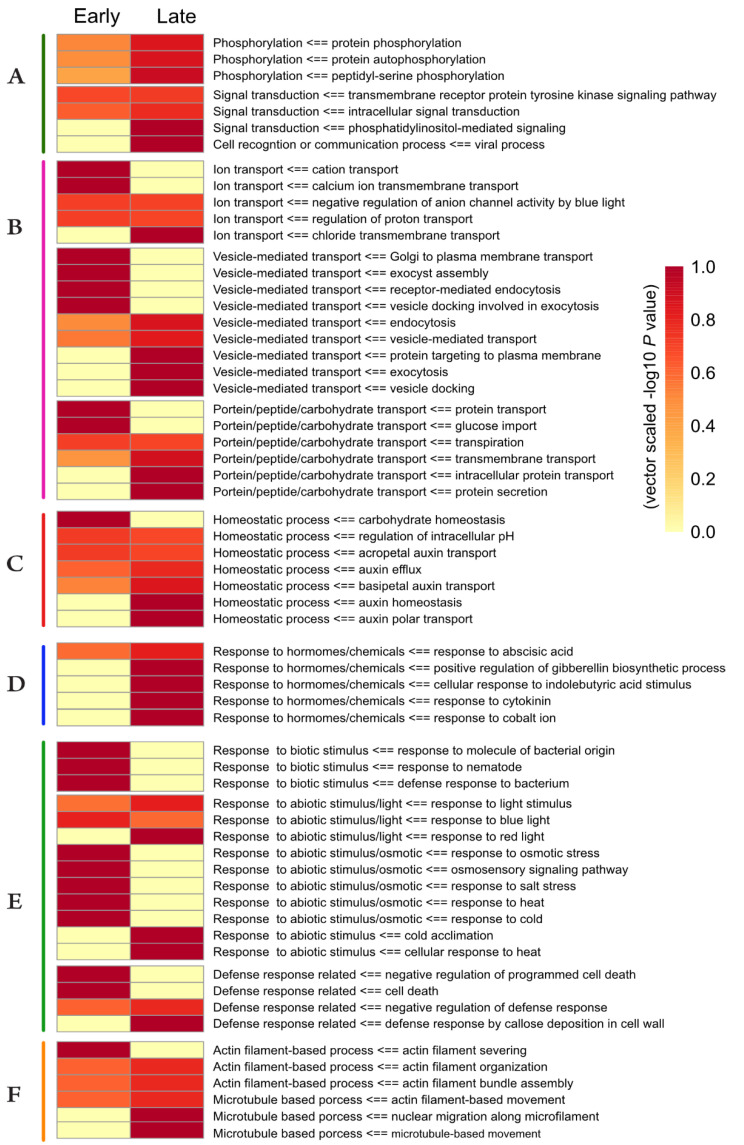
Gene ontology overrepresentation of biological processes (GOBP) for the phosphorylated proteins under cold exposure. Negative log 10 *p*-values of the GO terms enriched in the K-means clusters mentioned in Figure 2 were vector scaled and visualized. Sub-clusters (**A**–**F**) are based on the similarity of the GO terms extracted from ReViGO. Early, GO terms enriched within 5 to 15 min of cold exposure; Late, GO terms enriched within 30 to 60 min of cold exposure.

Changes in the molecular function of a gene product, such as protein or a group of proteins, in response to external or internal stimuli, initiate a collection of molecular events in different locations (cellular components) relative to the cell. These changes ultimately lead to a collective response from the cells, tissues, organs, and even the organism itself, contributing to a broader biological response/process [52,53]. To understand how the protein functionally changes due to phosphorylation upon cold exposure, we performed an over-representation analysis of the biological processes (GOBP) in early and late response clusters (Figure 4 and Figure S2). Proteins involved in phosphorylation and signal transduction were over-represented in both early and late response to cold exposure; however, these processes were highly abundant in the late response group compared to the early response group (Figure 4A). A large group of proteins involved in trafficking and transport-related events (ion transport, vesicle-mediated transport, protein/peptide, and carbohydrate transport) was differentially overrepresented in response to cold (Figure 4B), such as ion transport-related proteins phosphorylated in an early responsive manner, except for chloride ion transport. Vesicle-mediated transport-related proteins were over-represented throughout the cold exposure. However, proteins involved in Golgi-to-PM transport, exocyst assembly, exocytosis in vesicle docking, and receptor-mediated endocytosis were only enriched in the early response clusters. Interestingly, exocytosis, vesicle docking, and transport proteins targeting the PM were exclusively found in the late response clusters. Protein and glucose transport-related protein phosphorylation events took place during early cold exposure, but transmembrane transport, intracellular protein transport, peptide transport, and secretory protein transport-related processes were enriched in the late cold exposure. Auxin-related processes were most abundant in the late response clusters, including polar transport, basipetal transport, efflux, and auxin homeostasis. Proteins that play a role in maintaining homeostasis, such as carbohydrate homeostasis and pH regulation, responded to cold early (Figure 4C). Biological processes related to phytohormones, such as indole butyric acid, cytokinin, and gibberellic acid, were exclusive in the late response clusters, whereas the ethylene inhibitor, cobalt-ion-responsive phosphoproteins were only found in the early response clusters. Interestingly, the abscisic acid (ABA)-responsive proteins showed an increase in phosphorylation with cold exposure duration (Figure 4D). Proteins involved in responding to external stimuli, such as biotic stress and abiotic stress, including blue light, cold stress, and osmotic stress response, were significantly over-represented in the early response group. With a few exceptions, such as red light-responsive proteins, proteins involved in cold acclimation, and cellular response to heat, phototropism and gravitropism were found in the late response clusters (Figure 4E and Figure S2). Defense response-related protein phosphorylation was also abundant in the early response clusters, and interestingly cell death, negative response to cell death, and negative regulation of defense response-related proteins were highly enriched in the early response group. However, proteins responsible for CW callose deposition in response to stress were only found in the late response clusters (Figure 4E). Another interesting group of proteins phosphorylated under cold exposure belonged to the cytoskeleton/actin filament/microtubule-based process-related proteins. Most of the proteins from this cellular process were highly abundant in the late response clusters, except for the proteins involved the actin-filament severing process (Figure 4E). Proteins involved in other cellular/metabolic processes were mostly phosphorylated in late response to cold (Figure S2). In summary, brief temporal exposure to cold results in dynamic changes in a wide range of biological processes in *Arabidopsis.* These biological processes may be involved in early cold perception.

### 2.5. Cold-Responsive Phosphorylation Kinase Motifs

**Figure 5:**
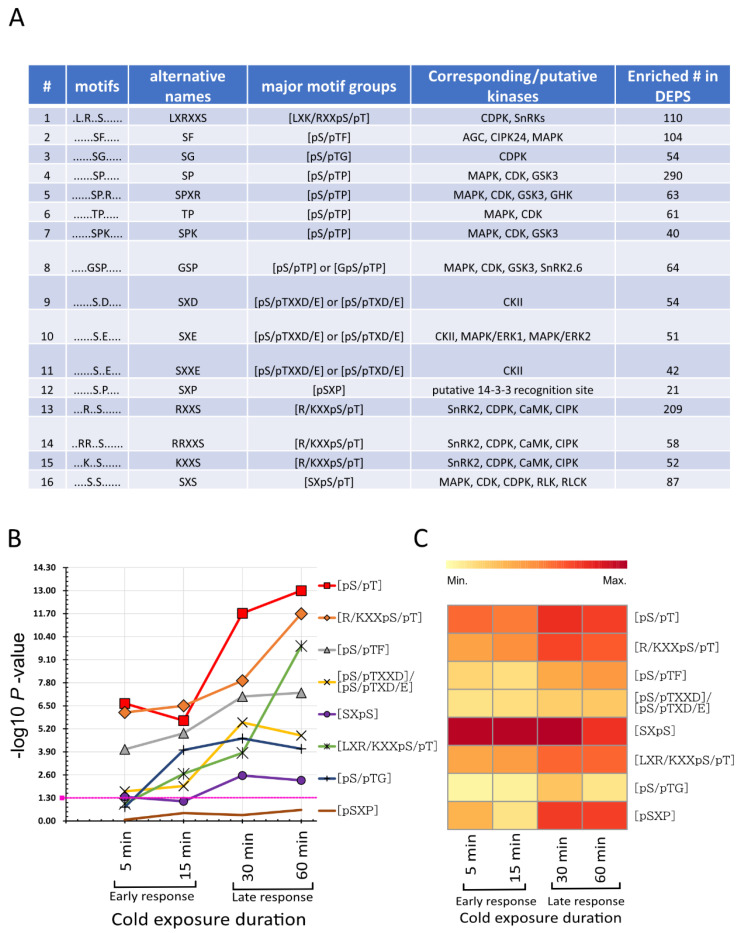
Cold-responsive phosphorylation kinase motifs. (**A**) Kinase motifs and major motif groups and their corresponding/putative upstream kinases. The numbers of corresponding motifs from the differentially expressed phosphopeptides (DEPS) are listed. (**B**) Temporal changes in the motif dynamics in response to cold. The *p*-values were obtained by Fisher’s exact test using enriched motif counts in DEPS and the major motif groups mentioned in (**A**). (**C**) Time-dependent change in the relative abundance in the corresponding phosphopeptides of each major motif.

We performed a motif analysis using all the differentially phosphorylated phosphosites (0.5 ≤ log_2_FC ≥ 1; *p* < 0.5) to gain insight into which kinases are involved in the early cold response. The Motif-x algorithm extracted 16 characteristic kinase motifs, where 15 were serine centered, and 1 was threonine centered, but no tyrosine-centered motifs were enriched (Figure 5A). These motifs were further grouped into eight major motif types, according to van Wijk et al. (Figure 5A) [54]. The SP or [pS/pTP]-type motif group in this study consisted of [pSP], [pTP], [GpSP], [pSPXR], and [pSPK] motifs. The RXXS or [R/KXXpS/pT] motif group contained [RXXpS], [KXXpS], and [RRXXpS] motifs. The [pS/pTXD/E] or [pS/pTXXD/E] motif group contained [pSXD], [pSXE], and [pSXXE] motifs. The other individually enriched motifs were [SXpS], [LXR/KXXpS/pT], [pS/pTF], [pS/pTG], and [pSXP]. Most of these motif sequences are well annotated to their up-stream kinases in the literature and databases [54,55,56,57,58,59,60,61,62,63]. Proline-directed motifs [pS/pTP] are a potential substrate for MAPK, cyclin-dependent protein kinase (CDK), CDK-like, and glycogen synthase kinase-3/shaggy-like kinase (GSK3/SLK). [R/KXXpS/pT] is a target motif for SNF1-related kinase II (SnRK2), calcium-dependent protein kinase (CDPK), Ca^2+^/calmodulin dependent protein kinase (CaMKII), and CBL interacting protein kinase (CIPK); it was also reported to be a binding motif for 14-3-3 proteins [61,62,63]. Another basic motif [LXR/KXXpS/pT] was also found to be targeted by CDPK, SnRK2s, and SnRK3s [58]. It was reported that SnRK2.6 targeted [GpS] motifs [58]. Interestingly, we also found the [GpSP] motif, which is similar to [GpS]. Even though we classified [GpSP] under the general [pS/pT] motif group, it might also be a potential recognition site for SnRK2.6. [SXpS/pT] has been reported to be recognized by MAPK, CDK, RLKs, and receptor-like cytoplasmic protein kinases (RLCKs) [54]. *Arabidopsis* AGC serine/threonine-protein kinase OXI1 (oxidative signal-inducible 1) and CIPK24/SnRK3.11 kinase SOS2 (salt overly sensitive 2) can recognize [pS/pTF], which is also known as a target for minimal MAPK [58]. The motif group [pS/pTXXD/E] or [pS/pTXD/E] is a target for casein kinase II (CKII), and [pSXE] is also a potential recognition site for plant ERK1 and ERK2 [50,64]. [pS/pTG] is a glycine-rich motif that mostly belongs to the secreted protein group and can be targeted by CDPK and protein phosphatase 2C (PP2C) [54]. Even though the [pSXP] motif has yet to be assigned to a specific kinase, it has been reported as a potential target site for 14-3-3 binding proteins [54]. Enrichment of these diverse groups of kinases indicates that brief exposure to cold significantly perturbed the kinome, and these potential kinases are involved in early cold-sensing and signaling networks. 

Next, we targeted each motif/motif group and assigned them to their corresponding source phosphopeptides to study the time-course based dynamics using Fisher’s exact test (Figure 5B). The motifs showed a characteristic response pattern to cold exposure. Even though few motifs were significantly enriched at all time points, their activity indicates the upstream kinase actions during cold exposure, which may affect the change in intensities in the corresponding target motifs (Figure 5C) [50,65]. In our data, we found that phosphopeptides containing [pS/pTP], [R/KXXpS/pT], [pS/pTF], and [pS/pTXXD/E]/[pS/pTXD/E] motifs were significantly enriched at all time points (Figure 5B). The phosphorylation tendency increased with cold exposure time, which was also visible from the increasing abundance of the phosphorylated peptides, indicating a higher level of kinase activity might belong to these motifs (Figure 5C). [SXpS] showed significantly increased enrichment at 30 and 60 min; however, the maximum abundance in the corresponding phosphopeptides was at 5 to 30 min with a decreasing trend at 60 min (Figure 5B,C). [LXR/KXXpS/pT] and [pS/pTG] showed increasing enrichment from 15 min, and the phosphopeptide abundance peaked in the late response clusters with an increase in the cold exposure period (Figure 5B,C). Even though the [pSXP] motif was not significantly enriched under cold exposure, its corresponding peptide abundance peaked at 30 and 60 min (Figure 5B,C).

### 2.6. Kinase–Substrate Interaction Network

**Figure 6:**
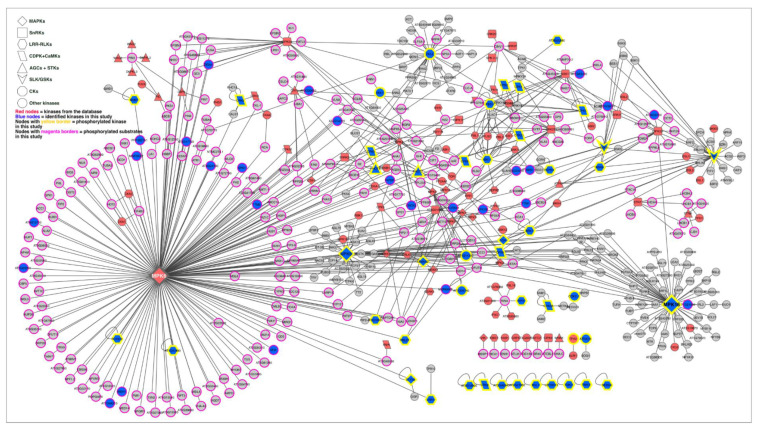
Cold-responsive kinase–substrate network in *Arabidopsis thaliana*. The network was constructed based on the identified kinase and substrates from this study and their corresponding interactions from the database-derived kinase–substrate interactions. The unique shapes of the nodes in this figure indicate a specific/unique group of kinases involved in the early cold response. Blue nodes indicate the kinases identified in this study; red nodes indicate the upstream kinases of the substrates identified in this study. Yellow and magenta borders indicate the phosphorylated kinases and substrates identified in this study, respectively.

We constructed an updated kinase–target network containing 3461 interactions (Figure S3 and Table S3A) based on the relevant databases and a literature search [45,55,56,57,58,65]. Differentially and significantly (*p* < 0.05) phosphorylated proteins in our study were then mapped into the kinase–substrate network to extract a subnetwork (Figure 6 and Table S3B). The interaction network revealed that early cold signaling involved 695 highly complex kinase–substrate interaction events among the MAPKs, SnRKs, CDPK/CaMKs, LRR-RLKs, SLKs, GSKs, CKs AGCs, and other kinases, and we also found their corresponding substrates in the motif-x analysis. Out of 695 interactions, MAP kinases, including MAPK, MAP2K, and MAP3Ks, formed nearly 350 interactions. ABA signaling-related kinases SnRKs, including SnRK1, SnRK2, and SnRK3, were involved in 95 interactions, followed by 59 interactions by LRR-RLKs, 45 interactions by CDPK and CaMK, 40 interactions by AGC and STKs, 38 interactions by SLK and GSKs, and 36 interactions by CKs and CKLs (Table S3C). The complex interactions between the different kinase–kinase and kinase–substrate counterparts indicate that the early cold response involves different signaling pathways to adjust to a low-temperature environment.

## 3. Discussion

This report provides dynamic evidence that signaling and molecular events in the model plant *Arabidopsis* switch by reversible protein phosphorylation after short exposure to cold. In this study, we found early cold response in plants at the posttranslational level associated with a complex network of cold signaling composed of various kinases and their targets. So far, to our knowledge, this is the first large-scale time-course phosphoproteomic study on *Arabidopsis* membranes and membrane-related proteins under brief cold exposure. This study will provide new insight into cold perception from a reversible phosphorylation point of view. 

### 3.1. Cold-Responsive Kinases and Signaling Proteins Might Play a Crucial Role in Early Cold Perception and Signal Transduction

Changes in phosphorylation to a large number of proteins in response to brief cold exposure indicate a significant perturbation in activity associated with kinases and phosphatases. A large number of enriched kinase target motifs from different major kinase families and direct identification of 152 kinases also imply a strong response from the kinases to cold exposure. 

Ca^2+^ is one of the essential signaling components in plants, and cytosolic Ca^2+^ changes have been reported as one of the first sensors of cold stress as well as other stresses [66,67,68,69,70]. Ca^2+^/calmodulin (CaM) binding, CaM/CaM-like proteins, and calcineurin B like (CBL) and CBL-interacting proteins (CIPKs) play significant roles in sensing and interpreting Ca^2+^ signals [71,72] as well as recruiting and activating other signaling components, such as RLKs, CDPK/CaMKs, MAPKs, and ROS, in response to cold stress [73]. PM-localized Ca^2+^/CaM-regulated receptor-like kinase 1 (CRLK1), a serine/threonine kinase, in *Arabidopsis* is reported to be involved in cold-stress response and activated by Ca^2+^/CaM [26]. Further studies showed that this CRKL1 could interact and phosphorylate MEKK1, a core component of the cold-regulated MAP kinase signaling pathway [25,29]. A phosphoproteomic study of cold stress in rice showed OsCDPK13 is an essential part of the cold response system [74]. CDPK phosphorylation, in response to chilling stress, was also reported in plants, including *Broussonetia papyrifera* and *Jatropha curcas* [75,76]. Similar to these studies, we also found several members of CDPK and CaM-domain protein kinase (CPK) (Table 1 and Table S1A) that were not reported to be regulated at the phosphorylation level in response to a brief cold response. In our study, a large number of CDPK/CaMK and CIPK target motifs ([LXR/KXXpS/pT], [R/KXXpS/pT], and [pS/pTG]) were enriched at the phosphorylation sites (Figure 5A) and in the kinase–target interaction network (Figure 6; Table S3B), indicating the role of the Ca^2+^-mediated signaling pathways, regulated at the phosphorylation level in the early cold response. 

Receptor kinases (RKs) or RLKs have been extensively studied at the phosphorylation level in response to pathogens and hormones. However, out of 600 RLKs, only about 60 have been functionally characterized so far [77]. Furthermore, only very few RLKs, for example, CRLK1 and RPK1, are functionally connected to cold response when exposed for more than several hours [25,29,78]. Thus, questions remain about whether any other RLKs respond to cold and how they respond at the PTM level. In this study, we found a large number of RLKs were phosphorylated in response to cold exposure (Table 1 and Table S1A). For instance, multifunctional somatic embryogenesis receptor kinase 4 (SERK4) is involved in brassinosteroid (BR) signaling and regulation of BR-dependent growth, and it also negatively regulates cell-death growth and phytohormone signaling [79,80,81,82]. Another peptide hormone-like receptor, RGF1 insensitive 3 (RGI3), is involved in meristem growth [83,84]. Transmembrane kinase 1 and 3 (TMK1 and TMK3) were reported to be involved in auxin-mediated cell growth and development and ROP GTPase signaling [85,86]. These RLKs might be involved in growth and development under cold stress. Botrytis-induced kinase 1 (BIK1) is one of the vastly studied RLKs that is central to the plant immune response and is also involved in Ca^2+^-dependent reactive oxygen (ROS) signaling, ethylene, and BR response [87,88,89], but it has never been reported to be phosphorylated in response to cold [87,88,89]. Receptor cytoplasmic kinase PBS1-like protein 27 (PBL27) activates the MAPK signaling cascade in response to chitin [90]. These findings imply that early RLK-mediated cold signaling might share, at least in part, some common pathways with other biotic, abiotic, ROS, and hormone signaling processes. Furthermore, the close phylogenetic relationship (Figure S4) between these cold-responsive RLKs could indicate a coordinated response from related gene family members at the PTM level under cold and could potentially be involved in cold perception and signaling in *Arabidopsis*. 

The most studied cold response pathway in *Arabidopsis* is the MAP kinase pathway, which is linked to freezing tolerance in cold-acclimated plants [23,27]. We have found four phosphoproteins from the poorly characterized MAP4K members of MAP kinases. Interestingly, three of these MAP4Ks were found to be closely positioned in the phylogenetic tree (Figure 5A)**.** Moreover, MAP4K5 and MAP4K6 were phosphorylated at the SXS motif in the central region of the protein but not inside any kinase domain. It could be possible that upstream RLKs or CDPKs phosphorylate these two proteins in the target SXS motif and regulate their function. It has been reported that some members of MAP4Ks can phosphorylate MAP3Ks or Raf kinases or directly phosphorylate MAP2Ks; others can function as adapters [91,92]. Similar to the MAP4Ks, phylogenetic analysis of the MAP3Ks and MAP3K-Raf (Figure 5B,C) showed most phosphorylated proteins are closely related to their gene family members, with a few exceptions, such as raf22, raf36, and raf47. For example, raf18 in this study, raf20, and raf24 [93], which are closely positioned in the phylogenetic tree, were phosphorylated within 5 to 30 min of cold exposure in the RXXS motif (Figure S5B). Even though there are no known functions ascribed to most Raf kinases, their response at the phosphorylation level to different stresses, including early cold (in this study) and early osmotic stress [42], indicates a potential involvement of Raf kinases in early cold-stress signaling. Other members of MAP3Ks phosphorylated in response to cold are also closely related in their phylogenies, such as M3KE1 and MAP3KE2, YDA/YODA, and MAP3Kγ (Figure S5C). These four MAP3Ks are reported to be involved in plant growth, development [94,95,96], and the MAPK signaling pathway [90,97]. Similar cold response phosphorylation patterns within identical phosphosites in closely related subfamily members of a large gene family could indicate coordinated phosphorylation and may be important for cold perception/signaling. Furthermore, phosphorylation of more downstream kinases, such as MKK2, MAPK6, MAPK8, and MAPK16, suggests that MAP kinases are involved in the early cold response and signal transduction. In summary, reversible phosphorylation of these MAP kinases, the highest enrichment in MAP kinase target motifs, and the highest number of kinase–substrate interactions indicate that the MAP kinase families play an essential role in early cold signaling. 

Enrichment of SnRK2 target motifs [R/KXXpS/pT] and [LXR/KXXpS/pT] as well as a large number of SnRK targets from the kinase–substrate network also indicate that the ABA signaling pathway is triggered in response to brief cold exposure. GOBP analysis showed ABA-responsive proteins were phosphorylated throughout the cold exposure (Figure 4D). Although we could not detect any SnRKs in our data, we found several SnRK substrates reported to be involved in different biological processes in response to different abiotic stresses, such as respiratory burst oxidase homolog protein D (RBOHD), ATP-binding cassette (ABC) protein G36 (ABCG36), plasma membrane intrinsic protein 2-7 (PIP2-7), phototropin 2 (PHOT2), syntaxins of plants 121 (SYP121), and myosin motor XI-K (Table 1, Tables S1 and S3). 

A few other important kinases and their substrates were also phosphorylated in response to cold exposure (Table 1 and Table S1), such as GSK3/shaggy-like kinase (ASK) brassinosteroid (BR) insensitive 2 (BIN2/ASK7), a positive regulator of BR signaling [98], and GSK3/SHAGGY-like protein kinase 1 (ASK9) and BSK4, negative regulators of BR signaling [99]. Phosphorylation of the BR signaling pathway regulators might indicate an involvement of BRs in early cold response in *Arabidopsis*. Casein kinase-like 2 (CKL2) was also phosphorylated immediately upon cold exposure. In addition, we found several reported and putative substrates of CK2 in the kinase–substrate network. A recent phosphoproteomic study in tomatoes also showed that CKL2 homologs were phosphorylated in response to prolonged cold stress, where SnRK2E could directly phosphorylate CKL2 [47]. 

### 3.2. Early Cold Response-Mediated Protein Phosphorylation Can Activate Phospholipid/Phosphoinositide Signaling Events

Phospholipids are the main building blocks of PM and responsible for maintaining the membrane structure and function. Phosphoinositides (PIs), one of the lipid family members present in small amounts in the membrane, are involved in various crucial functions in plant growth and development, including environmental adaptation. From the gene ontology analysis (Figure 4A), we found that phospholipid, phosphatidylinositol binding, and signaling-related proteins were phosphorylated within 15 min of cold exposure, indicating that phospholipids respond to the temperature drop at a very early stage. We found a total of 44 phosphopeptides from different lipid-related proteins, and 16 were involved in the phosphatidylinositol metabolism pathway (Table 1 and Table S1A). The phosphatidylinositol metabolism pathway members found in this study are involved in the generation and degradation of PIs. It has been reported that PIs are continuously generated and degrade rapidly and dynamically in response to different abiotic stresses such as wounding [100], heat stress [101], and salt stress or osmotic stress [102]. Phosphatidylinositol 4-kinase (PI4Ks) can phosphorylate PIs and generate PI monophosphates PI3P and PI4P. PI monophosphates can further be phosphorylated by forms aploid and binucleate1 (FAB1) and phosphatidylinositol 4-phosphate 5-kinase (PIP5K) into phosphatidylinositol 3,5-bisphosphate (PI(3,5)P_2_) and phosphatidylinositol 4,5-bisphosphate (PI(4,5)P_2_), respectively [103]. Vacuolar localization of PI(3,5)P_2_ under osmotic stress is essential for maintaining the proper structure and function of vacuoles in yeast [104,105,106]. PI(3,5)P_2_ also accumulates in the early osmotic stress response in *Arabidopsis* [42]. FAB1 and/or its product PI(3,5)P_2_ are essential for ABA-mediated stomatal closure, pollen tube growth, development, viability, endosome maturation, and interaction with microtubules [107]. In moss, PI(3,5)P_2_ are required for polarized cell growth and actin-cytoskeleton arrangement [108]. Endomembrane-localized *FAB1 kinase* knockout or knockdown results in enlarged vacuole formation in yeast because of impaired membrane trafficking [109,110,111]. The PI4P-5K product PI(4,5)P_2_ is also well known for its role in exocytosis, actin cytoskeleton organization, vesicle-mediated trafficking, and regulation of ion channels [112,113]. 

Furthermore, suppressor of actin 1 (SAC1) and phospholipase C/D (PLC/D) can degrade different PI species and generate downstream second messengers [103]. For example, PLC2 is one of the critical enzymes that can catalyze the hydrolysis of PI(4,5)P_2_ into diacylglycerol (DAG) and inositol 1,4,5-trisphosphate (IP3), which in turn increases Ca^2+^ influx into the cytoplasm [114,115]. Diacylglycerol kinases (DGKs) can convert DAG into phosphatidic acid (PA) [116], and PA is well known as a signaling molecule under different kinds of abiotic stresses, including cold. Interestingly, in our study, Sec14p-like phosphatidylinositol transfer family protein PATL3, responsible for transferring PIs to the PM [117], was found to be phosphorylated throughout the cold exposure duration (Table 1 and Table S1A). Phosphorylation of these PI kinases, phosphatases, and trafficking-related proteins clearly indicates that PI is involved in early cold perception, signaling, and cytoskeleton organization and might be regulated by posttranslational modifications.

### 3.3. Early Cold Response Events Can Be Associated with Cytoskeletal and Vesicle Trafficking Systems

The cytoskeleton in plants mainly consists of microtubules and actin filaments. It is essential for maintaining cell shape, and it facilitates organellar and vesicular trafficking inside the cell [118]. Several cytoskeleton-related proteins were phosphorylated in response to early cold exposure, including myosin and myosin-binding (MyoB) proteins myosin 1 (VIII-1), myosin-5 (XI-1), myosin-9 (XI-C), myosin-17 (XI-K), and MyoB7 (Table 1 and Table S1A). MyoB7 phosphorylation peaked at 5 min, and other myosin and myosin family proteins mostly peaked after 30 min of cold exposure. The myosin-binding protein MyoB1 showed increased phosphorylation within 2 min of osmotic stress [42]. In addition, myosin-like protein abundance was also found to be increased under cold stress in rice and perennial grasses [119,120]. Myosin and myosin-binding proteins facilitate cytoplasmic streaming [121]. Even though myosin and myosin-binding proteins respond to cold and osmotic treatments, their exact function still has to be determined. Perhaps, phosphorylation in the myosin-binding and myosin motor proteins could act as an early adaptive response to cold to maintain proper growth via cytoplasmic streaming and maintain cytoskeletal integrity of the cell under prolonged cold. 

Microtubule-related proteins, 65 kDa microtubule-associated protein 1 (MAP65-1), microtubule-associated protein 70-2 and 70-3 (MAP70-2 and MAP70-3), tubulin alpha-3 (Tuba-3), and microtubule-associated protein TORTIFOLIA1 (TOR1) were also phosphorylated and categorized in the late response clusters (30 min and 60 min, Table 1 and Table S1A). These proteins are responsible for the bundling and stabilization/destabilization of microtubules, the formation of tubulin dimers, and cell division [17]. Microtubules also act as sensors that perceive mechanical membrane stress derived from cold [122]. Furthermore, the sensory function of the microtubules is determined by dynamic instability and subsequent formation of stable microtubules during stress adaptation [122]. The MAP65-1 protein is phosphorylated in the microtubule-interacting region (pS532), and phosphorylation in this region disrupts the interaction properties [123]. Moreover, in the gene ontology analysis, we also observed that actin filament and microtubule-based processes were enriched in the late response group (Figure 4F). It might be possible that with the increasing duration and intensity of cold exposure, plant cells sense the cold via phosphorylation-mediated microtubule disruption, which acts as a cue for further cold adaptation-related processes.

Endosomal trafficking plays a crucial role in plant growth and development and maintains organellar homeostasis under different stressful conditions. Several studies showed endosomal transcript or protein abundance changes in response to longer durations (several hours to days) of cold stress [7,124,125,126]. However, before this study, it was unclear whether endosomes also respond to early cold exposure at the protein phosphorylation level. In our study, we observed changes in several previously described members of vesicle-mediated transport cargoes in plants at the phosphorylation level (Table 1 and Table S1A). For example, dynamin-like protein 2A and 2B (DRP2A, DRP 2B), sec7 domain-containing protein GN (GNOM), SYP121 and SYP132, soluble n-ethylmaleimide-sensitive factor adaptor protein 33 (SNAP33), sorting nexin 1 and 2A (SNX1, SNX2A), vacuolar protein sorting 41 (VPS41), and other proteins (Table 1 and Table S1A) showed cold-responsive phosphorylation within 60 min of cold exposure, suggesting that their PTM level changes might prepare plants for a longer cold exposure or stress. 

### 3.4. Transporters Respond to Early Cold Stress to Maintain Homeostasis 

Under any environmental stress situations, for proper growth, development, and survival, plants consistently need to maintain ionic and cellular homeostasis through different transporter and channel proteins located in the membranes by balancing the influx and efflux of the different ions, toxic byproducts, and carbohydrates [127]. Phosphorylation changes in the transporter proteins are also one of the rapid adaptive responses in plants under environmental stresses [37]. Many proteins related to solute and ion transport were also phosphorylated upon cold exposure (Table 1 and Table S1A). We found that two Ca^2+^ channel/transporters, the hyperosmolality-gated Ca^2+^ permeable channel 1.1 and 1.2 (AtOSCA1.1 and 1.2), were phosphorylated in the conserved C-terminal cytoplasmic domain at a close distance to each other at pT750 (at 15 min only) and pS757 (5 min and peaked at 30 and 60 min) (Table 1 and Table S1A). Both of these channels are known to permeate Ca^2+^ in response to osmotic stress, and AtOSCA1.1 is an essential component of osmosignaling [128,129]. However, these two phosphorylation sites were never functionally characterized in any stress response. Moreover, AtOSCA1.1 is also the first genetically identified Ca^2+^ channel. It is possible these two phosphosites, similar to osmosensing, play an essential role in sensing cold. Interestingly, we also found two autoinhibited Ca^2+^ ATPases (ACA), ACA1 and ACA4, were phosphorylated within 5 min and decreased gradually with the cold exposure duration (Table 1 and Table S1A). These ACAs are responsible for Ca^2+^ efflux out of the cell and maintaining the Ca^2+^ balance in the cell [130]. Elevation of the Ca^2+^ concentration inside the cell under cold is a significant signaling event that initiates further downstream canonical signaling pathways [68,69,70,131]. In this study, phosphorylation in both the Ca^2+^ influx and efflux channels is possibly involved in modulating a balanced Ca^2+^ level for fine-tuning Ca^2+^-mediated signaling events under cold. 

During the cold acclimation process, plants accumulate various cryoprotectants. Soluble sugars are one of the major cryoprotectants and can protect plants from freezing damage and maintain cellular integrity and functions [132]. In our study, we found several sugar and carbohydrate transmembrane transporters respond to early cold exposure, such as bidirectional sugar transporter SWEET12, polyol/monosaccharide transporter 5 (PLT5), tonoplast monosaccharide transporter 1 and 2 (TMT1, TMT2), sugar transporter ERD6, and ERD6-like 6 (ERDL6) (Table 1 and Table S1A). Reports showed disruption of SWEET12 resulted in thinner stems and reduced freezing tolerance in *Arabidopsis* [133], and its transcript levels increased under drought stress [134]. However, before this study, there was no report on the phosphoproteome level changes in SWEET12 in response to cold. Vacuolar monosaccharide transporters TMT1 and TMT 2 were reported to be phosphorylated at S385 and S376 under 5 days cold acclimation at 4 °C [135]. In our study, we found TMT1 was phosphorylated at pS277 throughout the cold exposure, and phosphorylation at pS446 immediately decreased at 5 min, then increased four-fold at 15 min and decreased at 30 and 60 min. TMT2 showed increased phosphorylation at pS448 peaked at 5 min and was maintained throughout the cold exposure, where pS287 peaked at 60 min only (Table 1 and Table S1A). All phosphorylation took place between transmembrane domains 5 and 6, and 6 and 7, which generally agrees with in silico prediction studies of the phosphorylation sites in TMTs [136]. Similar to the reports showing that phosphorylation regulates sugar transport under prolonged cold stress and other abiotic stresses [135], our study indicates that, in *Arabidopsis*, the early cold response also initiates sugar transport-related events in the PM and vacuoles and might be regulated by phosphorylation. 

We observed strong phosphorylation in two PM proton-pump members: H^+^ATApase1 (AHA1) and H^+^ATPase4 (AHA4) (Table 1 and Table S1A). AHA1 was phosphorylated at multiple sites, including the canonical 14-3-3 protein binding YTV domains such as T881, T948, and the inhibitory S899 site. AHA4 showed constant phosphorylation at pT959 inside the 14-3-3 protein binding YTV domain throughout the cold exposure after peaking at 5 min. The binding of 14-3-3 protein at pT948 and pT959 in the YTV domain activates the proton pump [137,138]. On the other hand, phosphorylation at pS899 inhibits pump activity by an unknown mechanism [139]. The observed phosphorylation at both the 14-3-3 protein binding sites and the pS899 inhibitory site in this study seem to be contradictory to the cold response. Even though AHA1 is phosphorylated at the inhibitory pS899 site, the pumps might be hyperactivated by the phosphorylated interactor isoform 1 (PPI1). It has been reported that PPI1 can hyperactivate AHAs [140]. We observed immediate phosphorylation of PPI1 at 5 min, and increased phosphorylation was maintained at 30 min and 60 min (Table 1 and Table S1A). 

One of the most potent transporter groups in plants is the ATP-binding cassette (ABC) transporters. ABC transporters are reported to be involved in sequestration of toxic byproducts and in exchanging compounds like phytohormones (auxin), secondary metabolites, and defense molecules [141,142]. ABC transporters have mostly been studied at genomic and transcriptomic levels; for example, plants with overexpressed ABCG36 show improved resistance to salt and drought stress [143]. ABCC1 acts as a pump for glutathione S-conjugates [144], and ABCB1, ABCB14, ABCB19, ABCG36, and ABCG37 were reported to be involved in auxin transport-mediated plant growth and development [145,146,147]. In addition, a few studies showed their regulation and function at the phosphorylation level; for example, ABCB19 is a direct phosphorylation target of kinase PHOT1, which can transiently deactivate ABCB19 and result in the accumulation of auxin [148], and ABCG36 is phosphorylated in response to pathogen attack. We also found several ABC transporters to be phosphorylated under early cold exposure, and 75% of these were immediately phosphorylated within 5 min of cold exposure (Table 1 and Table S1A). The most notable transporters found in our study were ABCG36/37, ABCB1, and ABCC1, which were involved in detoxification, auxin transport, biotic and abiotic stress responses, and resistance [141]. The PTM or phosphorylation-level regulation of these ABC transporters found in this study might play a crucial role in maintaining homeostasis and detoxification as an adaptive early cold response in *Arabidopsis*.

Auxin is a key phytohormone involved in different regulatory processes in plants. The spatiotemporal distribution of auxin is critical for auxin-mediated growth and development under normal and stressful conditions, including cold [149,150,151]. Studies reporting the involvement of auxin transport or homeostasis are mostly carried out under prolonged exposure (several hours to days), but not short (within minutes) exposure to stress as we employed in our study. Furthermore, most studies have focused on functional regulation at the genomic or transcriptomic level than the PTM level. Reversible phosphorylation at conserved serine and threonine residues could control the spatiotemporal distribution of auxin efflux carrier family protein 1 (PIN1) and its transport activity [152]. In our study, we found auxin transporter proteins, such as AUX1/LAX (influx) and PIN3, 4, and 7 (efflux), were phosphorylated in response to early cold stress (Table 1 and Table S1A). AUX1 was phosphorylated immediately and maintained its phosphorylation throughout the cold exposure time at two different phosphosites, pS14 and pS27, and inside the target SXXD/E motifs for casein kinase (CKII). On the other hand, PIN3 was phosphorylated at conserved pS366 with a 1.8-fold increase at 5 min and peaked at 60 min. PIN4 was also phosphorylated at conserved pS395 within the SXXD/E motif and maintained its phosphorylation throughout the cold exposure/treatment time and peaked at 60 min. We also found a phosphosite of PIN7 at pS431 in the SXXD/E motif, which maintained a continuous phosphorylation state. CKII was also reported to play an essential role in auxin transport and auxin-mediated growth and development in *Arabidopsis* [153,154]. Interestingly, PIN7 was also phosphorylated at T242 in the conserved TPRXSN motif. The TPRXSN motif is involved in phosphorylation-dependent control of PIN1 localization [152]. Cold-responsive phosphorylation of PIN and AUX1 at different conserved sites indicates an essential role of protein phosphorylation under a short period of cold exposure, which might be involved in auxin transport and homeostasis for maintaining growth and development for upcoming longer and extreme cold conditions. 

Our data also showed that PM intrinsic proteins (PIPs) respond actively and temporally to brief cold exposure (Table 1 and Table S1A). We observed continuous phosphorylation of PIP2-5 (pS279) throughout the cold treatment. A doubly phosphorylated peptide of PIP2-6 (pS279 and pS282) was phosphorylated over two-fold at 60 min, and PIP2-7 (pS276) showed decreasing phosphorylation after peaking at 5 min of cold exposure (Table 1 and Table S1A). PIPs are transmembrane transporters for water and small molecules and operate in a gated, controlled way. Gating properties vary under different abiotic stresses and are controlled by pH in flooding/anoxic conditions [155,156], phosphorylation/dephosphorylation under drought stress and low temperature [157,158], and calcium under salt stress [159]. It was also reported that chilling-induced H_2_O_2_ could be diffused by PIPs in a phosphorylation-dependent manner in maize [160]. Previously, it was shown that phosphorylation at pS280 and pS283 of PIP2-1 was associated with gating or hydraulic conductivity [161]. Multiple sequence alignment analysis showed that pS279 of PIP2-5 and PIP2-6, and pS276 of PIP2-7, are conserved with pS280 of PIP2-1, and the pS282 of PIP2-5 is also conserved with pS283 of PIP2-1 (data not shown). Phosphorylation of PIP2s in all these phosphosites suggests that the activity of the PIPs is controlled in a phosphorylation-dependent manner under cold and maintains cellular water and H_2_O_2_ in a balanced state. Previous studies also showed that PIP1-4 and PIP2-5 could regulate cold acclimation and freezing tolerance in *Arabidopsis* [162]. Thus, continuous phosphorylation of PIP2-2 at its regulatory S279 position throughout cold exposure, along with other PIP2s, might play a crucial role in cold response and prepare plants for long-term cold acclimation.

### 3.5. ROS Signaling under Cold Response

ROS, generating respiratory burst oxidase homolog D (RBOHD), were also phosphorylated, as shown in this study, which has been never reported in response to brief cold exposure (Table 1 and Table S1A). In this study, RBOHD was phosphorylated in pS8, pS148, pS163, and pS347 within 15 min of cold exposure (Table 1 and Table S1A). RBOHD can generate ROS via Ca^2+^-dependent and -independent phosphorylation by CDPKs/CIPKs and BIK1 kinase, respectively [87,163]. Ca^2+^-dependent protein kinase 5 (CPK5) and BIK can phosphorylate and regulate RBOHD at the same amino acid positions (pS148, pS163, and pS347) [163,164,165], which are the same positions we observed as the cold-responsive phosphorylated sites in this study. Interestingly, in our data, we detected that both CPK5 and BIK were phosphorylated in response to brief cold (Table 1 and Table S1A). Furthermore, the phosphosites showed increased phosphorylation containing characteristic RXXS/LXRXXS motifs, a potential target motif of CDPKs (Table 1 and Table S1A). This rapid phosphorylation of ROBHD and its kinases indicates ROS signaling involvement in early cold response. 

## 4. Materials and Methods 

### 4.1. Plant Growth Conditions and Cold Exposure

Twenty-five surface-sterilized seeds of *Arabidopsis thaliana* ecotype Colombia-0 (Lehle Seeds, Round Rock, TX, USA) were sown in 9 cm square petri dishes containing modified Hoagland solution [166] with 0.8% agar (Wako, Tokyo, Japan). The seeds were then cold stratified for 48 h at 4 °C. The seeds were germinated and grown for two weeks at 23 °C and a 16/8 h photoperiod at 80–100 μmolm^−2^s^−1^ light intensity in a growth chamber (CLE-303, Tomy Digital Biology, Tokyo, Japan). For cold exposure analysis, agar plates containing two-week-old *Arabidopsis* seedlings were exposed to 2 °C for 0 min (control: no cold exposure), 5 min, 15 min, 30 min, and 60 min without the lid of the petri dish. Plates were flash-frozen in liquid nitrogen at the end of each cold exposure duration. The frozen plants were used for protein extraction or stored at −80 °C for later use. For each time point three independent biological replicates were used. 

### 4.2. Microsomal Membrane Fraction (MMF) Extraction

MMF was extracted according to the method of Wu et al. [45], with modifications. Around 75 two-week-old pre-frozen seedlings (~1–1.5 g) were broken into small pieces and homogenized in 10 mL of homogenization buffer consisting of 500 mM sorbitol, 50 mM 3-(n-morpholino) propanesulfonic acid-KOH (MOPS-KOH) (pH 7.6), 5 mM ethylenediaminetetraacetic acid (EDTA), 5 mM dithiothreitol (DTT), 1 mM phenylmethylsulfonyl fluoride (PMSF), 2 mM salicylhydroxamic acid (SHAM), 25 mM sodium fluoride (NaF), 0.5 × protease inhibitor cocktail (p9599, Sigma-Aldrich, St. Louis, MO, USA), and 1 × phosphatase inhibitor cocktail 2 and 3 (P5726 and P0044, Sigma-Aldrich, St. Louis, MO, USA), using a 30 mL motorized glass homogenizer (81-0487, Sansyo Co., Ltd., Tokyo, Japan) on ice. For proper homogenization, the rotatory pastel was moved up and down slowly for at least 3 min. The homogenate was filtered through six layers of cheesecloth and centrifuged at 7500× *g* for 15 min at 4 °C. The supernatant was collected and ultracentrifuged at 100,000× *g* for 50 min at 4 °C. The pellet was washed twice with homogenization buffer by centrifugation at 100,000× *g* for 20 min at 4 °C. The final washed pellet was collected as MMF and resuspended in a minimum volume of Tris-UTU buffer (100 mM tris aminomethane, 6 M urea, 2 M thiourea, pH 8.5). The protein concentration was measured using a Bradford (Bio-Rad, Hercules, CA, USA) assay [167]. The MMF samples were used for peptide processing immediately or stored at −80 °C for later use. 

### 4.3. Phosphoproteomic Analysis

#### 4.3.1. In-Solution Trypsin Digestion 

The phosphoproteomic analysis was carried out according to methods previously reported [44,46,49,50,168,169], with minor modifications. For trypsin digestion, aliquots of MMF (1 mg protein) were used. The digested MMF was diluted four times with sterile distilled water, reduced with 10 mM DTT for 60 min, and then alkylated in the dark with 55 mM iodoacetamide for 30 min at room temperature. After the alkylation, the proteins were digested overnight at room temperature with trypsin gold (1:100, w/w; Promega, Madison, WI, USA). After digestion, the sample tubes were centrifuged for 5 min at 21,000× *g* at room temperature. The supernatant was transferred to a fresh tube and used for phosphopeptide enrichment. 

#### 4.3.2. Phosphopeptide Enrichment and Desalting

Phosphopeptides were enriched at room temperature using hydroxy acid-modified metal oxide chromatography (HAMMOC), with minor modifications [168]. Titansphere^®^ Phos-TiO columns (syringe barrel type SPE cartridge, cat. No. 5010-21291, GL Sciences Inc., Tokyo, Japan) were used according to the manufacturer’s instructions. Enriched phosphopeptides were desalted using two-step washing with a combination of GL-Tip SDB and GC columns (GL Sciences Inc., Tokyo, Japan), according to the manufacturer’s instructions. Desalted phosphopeptides were dried in a vacuum evaporator (Tomy, Tokyo, Japan), resuspended in 10 µL of 0.1% (*v*/*v*) TFA, and stored at −80 °C for later LC-MS/MS analysis.

#### 4.3.3. LC-ESI-MS/MS Analysis

Desalted phosphopeptides were analyzed in the TripleTOF5600 system (AB SCIEX, Framingham, MA, USA) equipped with Autosampler-2 1D plus (Eksigent, Framingham, MA, USA) and NanoLC Ultra (Eksigent, Framingham, MA, USA) using a MonoCap C18 High-Resolution 2000 column (GL Sciences Inc., Tokyo, Japan) and PicoTip emitter SilicaTip (New Objective Inc., Woburn, MA, USA). Peptides were eluted at a flow rate of 500 nL/min^−1^ with a four-step gradient using 0.5% (*v/v*) acetic acid: 0.5% (*v/v*) and 80% (*v/v*) acetic acid = 98:2 (0 min), 60:40 (300 min), 10:90 (20 min), and 98:2 (40 min). The eluate was sprayed into the mass spectrometer by electrospray ionization (ESI). The mass spectrometry (MS) scan range was 400–1250 *m/z*, and the MS/MS scan range was 100–1600 *m/z*.

#### 4.3.4. Phosphopeptide Identification and Quantification

The spectrum files (wiff and wiff.scan) generated from the mass spectrometer were converted to peak lists in mascot generic format (mgf) using Protein Pilot software (v5.0.0.4769; AB-SCIEX, Framingham, MA, USA). The mgf files were searched against the *Arabidopsis* protein database Araport11 (11 July 2019) [170] obtained from the *Arabidopsis* information resource (TAIR) (https://www.Arabidopsis.org/) [171] in the Mascot ion search platform (v2.6, Matrix Science, London, UK). The search parameters were applied as follows: a precursor mass tolerance of 3 ppm, a fragment ion mass tolerance of 0.8 Da, and a cut-off value of 0.95, allowing for up to two missed cleavage from trypsin digestion. Fixed modification of carbamidomethylation of cysteine and variable modifications of oxidation of methionine and phosphorylation of serine, threonine, and tyrosine were used [44,46,50,169,172]. The false discovery rate (FDR) was calculated using the Benjamini–Hochberg method and set to 5%. The phosphorylation site localization confidence was assessed by site localization probability scores calculated from the mascot delta score, and the threshold level was set to > 0.75 to identify class-I phosphorylation sites [46,173].

Skyline-Daily software (v20.1.1.83; https://skyline.ms/project/home/software/Skyline/daily/begin.view) [174] was used to quantify the peak areas/intensities. The search parameters in the Skyline-Daily were the same as those in the Mascot search parameters. A minimum of two peptides identified per protein was considered as a confident identification of that protein. The peak intensities, from both the phosphorylated and proteotypic non-phosphorylated peptides generated from Skyline-Daily, were normalized using the EigenMS normalization method [175,176,177] in the ProteoMM R-Bioconductor package [178]. All raw data files were deposited in the Japan Proteome Standard Repository/Database (jPOST; JPST000993, Kyoto, Japan).

### 4.4. Bioinformatic Analysis

Identified phosphopeptides and proteotypic non-phosphopeptides from all biological replicates from each time point (0, 5, 15, 30, and 60 min) were compared using unsupervised principal component analysis (PCA). The phosphopeptides identified in all five conditions were selected for further analysis. The changes in phosphorylation dynamics were studied by K-means clustering of the log-transformed normalized phosphopeptide intensities in MeV (MultiExperiment Viewer), using Euclidean distances [179]. Comparative analysis between the untreated (0 min) and treated (cold exposed) groups was performed in each characteristic K-means clusters; increased phosphorylation was assigned if the log_2_ fold change (Log_2_FC) of the treated/untreated ration was ≥ 1 in at least two of the three biological replicates for each time point, and a decreased phosphorylation was when the Log_2_FC was ≤ 0.5 in at least two of three replicates. Individual phosphopeptides were compared using pairwise t-tests between the average intensities at 0 min and each cold exposure time point. The relative time profile for significant phosphopeptide changes in each cluster was also created based on the Log_2_FCs between 0 min and all other cold exposure time points. All time-profile clusters were visualized using a heatmap in the online platform ClustVis (https://biit.cs.ut.ee/clustvis/) [180].

#### 4.4.1. Gene Ontology Analysis

Gene Ontology (GO) annotation (GO-Slim, last update: 1 May 2020) for all *Arabidopsis* proteins was obtained from the latest TAIR [171]. The GO annotation database was created by excluding genes with gene ontology-consortium evidence code IEA (Inferred from Electronic Annotation) and ND (No biological Data available) for further analysis. Over-representative GO term analysis was carried out in AgriGO (v2.0: http://systemsbiology.cau.edu.cn/agriGOv2/) [181,182], uploading the GO annotation database as background. The Singular Enrichment Analysis (SEA) tool was used in the AgriGO platform combined with Fisher’s exact test and Yekutieli’s test (FDR under dependency) for the multi-test *p*-value (0.05) adjustment method; the minimum number of mapping entries was five, and plant GO-Slim was the Gene Ontology type. To decrease the chance of false discovery, GO cross-validation was also carried out in DAVID: BP_Direct (v6.8: https://david.ncifcrf.gov/) [183,184] and CluterProfiler: groupGO function [185] using the same updated GO annotation database used in AgriGO as background, in g:Profiler g:GOSt using *Arabidopsis* as background and g:SCS threshold 0.05 [186], and finally matched with SUBA4 localization [187]. Significantly (adjusted *P*-value ≤ 0.05) enriched GO cellular component (GO_CC) terms related to MMF, such as plasma membrane (PM), endoplasmic reticulum (ER), Golgi (G), vacuole (V), cell wall (W), apoplast (A), cytoskeleton (CS), plasmodesma (PD), and other membrane-related proteins, were selected as the protein group of interest for further analysis. Proteins that belong to the cytosol (C), plastid (P), mitochondria (M), and nucleus (N) were excluded from further analysis.

For the over-representation analysis of GO biological processes (GOBP) and molecular function (GOMF), only the protein group of interest was used in process similar to that for GOCC. Significantly over-represented GO terms (BH adjusted *p* < 0.05 after Fisher’ exact test) were grouped in similar categories using REViGO (http://revigo.irb.hr/) [188]. Settings used in REViGO were as follows: medium (0.7) similarity, UniProt *Arabidopsis* database (https://www.uniprot.org), and SimRel semantic measure. The negative log10-transformed, adjusted *p*-value < 0.05 for each GO term in the REVIGO groups was scaled with vector scaling and visualized in a heatmap in the online platform ClustVis (https://biit.cs.ut.ee/clustvis/) [180].

#### 4.4.2. Phosphorylation Motif Dynamics Analysis

Phosphorylation motifs were predicted using the motif-x [189] algorithm in rmotifx (https://github.com/omarwagih/rmotifx) [190] in the R package. All confident phosphopeptides were used for motif prediction. Phosphopeptide sequences were centered around the phosphorylation sites (STY), and 13 amino acids (±6) around the phosphorylated residues were extracted and submitted to the rmotifx. In cases where the phosphorylation sites were near the N/C terminal, the motif sequences were filled in using 13 amino acids with the required number of “X”, which denotes any amino acid [65]. The settings for rmotifx were as follows: the minimum number of central amino acid residues was set to 30 for Ser (S) and 20 for Thr (T) and Tyr (Y), the significance threshold was set to *p* < 10^−6^, and background database was extracted from Araport11 [170]. Any enriched motif was attributed to a kinase or kinase group if the identical motif was reported in the literature or matched in the PhosPhAt database [55,56,57]. Similar motifs were grouped according to van Wijk et al. [54].

To predict kinase activity and kinase regulation dynamics, each enriched motif was assessed at each time point. The activity of upstream kinases affects the phosphorylation intensity of the downstream target motifs. To reveal the effect of kinases on targets, we computed the total average intensities from the phosphopeptides that belong to each enriched motif or motif groups at each time point and calculated the fold change between 0 min and any other cold exposure time point [50]. Motif dynamics were assessed using Fisher’s exact test using a 2 × 2 contingency table, as described by Lin et al. [65].

#### 4.4.3. Kinase–Substrate Network Construction

An updated kinase–substrate network was built by collecting experimental data from the literature [58] and the PhosPhAt database [55,56,57]. The constructed network was used as a benchmark for mapping and visualizing the identified kinases and putative substrates in this study by Cytoscape [191].

## 5. Conclusions

In summary, to understand phosphoregulation in the earliest stage of cold response (within minutes), we performed a large-scale mass spectrometry-based phosphoproteomic analysis of the *Arabidopsis* microsomal membrane proteins. We detected nearly 1900 phosphosites from over 850 proteins, where around 1000 phosphosites showed temporal phosphorylation in response to cold. From an in-depth analysis of the phosphoproteomic data, we revealed that a plant’s response to a brief drop in temperature is a rather complicated process. Phosphorylation of ion channel or transporter proteins, such as Ca^2+^ (OSCAs, ACAs), K^+^ (POTs), or Na^+^/H^+^ (NHXs), which are involved in maintaining ionic homeostasis inside the cell, indicated that plant cells try to maintain ionic balance in a steady state under cold conditions. Our data also indicated that the plant kinome is perturbed by cold exposure and kinases, such that RLKs, MAPKs, CDPKs/CaMKs, CKs, and GSKs play active roles in cold sensing and signaling processes. Ca^2+^, phosphoinositide, ABA, auxin, BR, and ROS mediated signaling pathways were also found to be involved in the early cold response. Protein transport, trafficking, and cytoplasmic streaming may also be involved in early cold response events. We further found that plant cells tried to maintain cytoskeletal integrity as an adaptive response to cold, which was seen by the rapid phosphorylation of proteins involved in actin-/microtubule-based processes. We expect that this first large-scale membrane phosphoproteomic study to elucidate early cold response in *Arabidopsis* will provide valuable information to understand cold perception in plants.

## Figures and Tables

**Table 1:** The phosphopeptides described in the Results and Discussion sections.

TAIR Accession	TAIR Symbol	Protein Description	Localization	Bin Names of Functional Categories	Phosphopeptides	Phospho-residue	Phospho-sites	KMC Clusters
AT1G59610	DRP2B	dynamin-like 3	PM	Vesicle trafficking	RYS[+80]DPAQNGEDSSGSGGSSR	S	883	E,C *
AT1G10290	DRP2A	dynamin-like protein 6	G,PM	Vesicle trafficking	RYS[+80]DPAQNGDAASPGSGSNRR	S	877	A *
AT1G13980	GN	sec7 domain-containing protein	G,PM	Vesicle trafficking	FSQLLS[+80]LDTEEPR	S	961	C *
AT5G06140	SNX1	sorting nexin 1	C,PM	Vesicle trafficking	NISGSMQS[+80]PR	S	16	C *
AT3G11820	SYP121	syntaxin of plants 121	PM	Vesicle trafficking	TLDRLIS[+80]TGESER	S	190	C *
AT5G08080	SYP132	syntaxin of plants 132	PM	Vesicle trafficking	GQS[+80]SREGDVELGEQQGGDQGLEDFFKK	S	16	D *
AT5G61210	SNAP33	soluble N-ethylmaleimide-sensitive factor adaptor protein 33	PM	Vesicle trafficking	TTS[+80]EPSLADMTNPFGGER	S	47	E,C *
AT5G58440	SNX2A	sorting nexin 2A	G,EM	Vesicle trafficking	SPS[+80]SSSSDYIK	S	146	A *
AT1G08190	VPS41	vacuolar protein sorting 41	G,EM	Vesicle trafficking	EDNNRSS[+80]FSQR	S	860	B *
AT5G06560	MYOB7	myosin-binding protein (Protein of unknown function 2C DUF593)	EM	unknown	FKNDTADGYAMS[+80]PR	S	385	E,A *
AT4G27500	PPI1	proton pump interactor 1	ER,PM	unknown	KKTGGNTETETEEVPEAS[+80]EEEIEAPVQEEKPQK	S	540	E,C *
AT1G72160	PATL3	Sec14p-like phosphatidylinositol transfer family protein	PM	Solute transport	SMIPQNLGS[+80]FKEESSKLSDLSNSEK	S	108	E,C *
AT2G41560	ACA4	autoinhibited Ca^2+^-ATPase 2C isoform 4	V,PM	Solute transport	SSVS[+80]IVKNR	S	28	A *
AT1G27770	ACA1	autoinhibited Ca^2+^-ATPase 1	PM	Solute transport	FTANLS[+80]KR	S	46	A *
AT4G04340	AtOSCA1.1	*ERD* (early-responsive to dehydration stress) family protein	PM	Solute transport	RNT[+80]PAPSR	T	750	B *
AT4G22120	AtOSCA1.2	*ERD* (early-responsive to dehydration stress) family protein	PM	Solute transport	NTPAPSIIS[+80]GDDSPSLPFSGK	S	757	C *
AT5G23660	SWEET12	bidirectional sugar transporter *SWEET12*-like protein	PM	Solute transport	LGTLTS[+80]PEPVAITVVR	S	248	A *
AT1G08930	ERD6	Major facilitator superfamily protein	ER,PM	Solute transport	SLS[+80]IRER	S	17	A *
AT3G18830	PLT5	polyol/monosaccharide transporter 5	PM	Solute transport	TVPNPEVEIGS[+80]NKQWKEGDTQSS	S	527	D *
AT1G20840	TMT1	tonoplast monosaccharide transporter1	V,PM	Solute transport	LYGTHENQSYLARPVPEQNS[+80]SLGLR	S	277	E,A *
AT1G20840	TMT1	tonoplast monosaccharide transporter1	V,PM	Solute transport	YYLKEDGAES[+80]R	S	446	B*
AT4G35300	TMT2	tonoplast monosaccharide transporter2	V,PM	Solute transport	IYLHQEGFPGS[+80]RR	S	448	E,A *
AT4G35300	TMT2	tonoplast monosaccharide transporter2	V,PM	Solute transport	HGS[+80]TMSR	S	287	D *
AT1G75220	ERDL6	Major facilitator superfamily protein	V,PM	Solute transport	RPFIHTGS[+80]WYR	S	23	D *
AT2G18960	AHA1	H[+]-ATPase 1	PM	Solute transport	T[+80]LHGLQPKEDVNIFPEKGSYR	T	881	D *
AT2G18960	AHA1	H[+]-ATPase 1	PM	Solute transport	GLDIDTAGHHYT[+80]V	T	948	D *
AT2G18960	AHA1	H[+]-ATPase 1	PM	Solute transport	EDVNIFPEKGS[+80]YRELSEIAEQAK	S	899	E,D *
AT3G47950	AHA4	H[+]-ATPase 4	PM	Solute transport	GLDIETIQQAYT[+80]V	T	959	E,A *
AT3G54820	PIP2-5	plasma membrane intrinsic protein 2%3B5	PM	Solute transport	ALGS[+80]FRSQPHV	S	279	E,D *
AT2G39010	PIP2-6	plasma membrane intrinsic protein 2E	PM	Solute transport	AYGS[+80]VRS[+80]QLHELHA	S;S	279;282	D *
AT4G35100	PIP2-7	plasma membrane intrinsic protein 3	PM	Solute transport	ALGSFRS[+80]NATN	S	276	C *
AT2G36910	ABCB1	ATP binding cassette subfamily B1	PM	Solute transport	NSVSS[+80]PIMTR	S	634	A *
AT1G30400	ABCC1	multidrug resistance-associated protein 1	V,PD	Solute transport	SIT[+80]LENKR	T	1485	A *
AT1G59870	ABCG36	ABC-2 and Plant PDR ABC-type transporter family protein	PM	Solute transport	SLS[+80]TADGNRRGEVAMGR	S	825	B *
AT3G53480	ABCG37	pleiotropic drug resistance 9	PM	Solute transport	MNLS[+80]YWR	S	1187	C *
AT5G01240	LAX1	like AUXIN RESISTANT 1	PM	Solute transport	QAEESIVVS[+80]GEDEVAGR	S	14	E,A *
AT5G01240	LAX1	like AUXIN RESISTANT 1	PM	Solute transport	KVEDS[+80]AAEEDIDGNGGNGFSMK	S	27	B *
AT5G47910	RBOHD	respiratory burst oxidase homologue D	PM	Redox homeostasis	VFS[+80]RRPSPAVR	S	148	B *
AT5G47910	RBOHD	respiratory burst oxidase homologue D	PM	Redox homeostasis	TSS[+80]AAIHALKGLK	S	163	C *
AT5G47910	RBOHD	respiratory burst oxidase homologue D	PM	Redox homeostasis	ILSQMLS[+80]QK	S	347	B *
AT3G15220	AT3G15220	Protein kinase superfamily protein	MT, CT	Protein modification	RQEVS[+80]PNRISQR	S	364	D *
AT5G14720	AT5G14720	Protein kinase superfamily protein	PM	Protein modification	YLEQTSAKQPGS[+80]PETNVDDLLQTPPATSR	S	613	E,C *
AT4G24100	AT4G24100	Protein kinase superfamily protein	PM	Protein modification	SDS[+80]NGNVEPVASERER	S	654	E,D *
AT4G10730	AT4G10730	Protein kinase superfamily protein	PM	Protein modification	KSAS[+80]VGNWILDSK	S	579	C *
AT1G63700	YDA	Protein kinase superfamily protein	PM	Protein modification	S[+80]LPCLDSEDATNYQQK	S	692	D *
AT5G66850	MAPKKK5	mitogen-activated protein kinase kinase kinase 5	PM	Protein modification	SPS[+80]AFTAVPR	S	90	C *
AT3G07980	MAP3KE2	mitogen-activated protein kinase kinase kinase 6	V	Protein modification	KIS[+80]GQLDYVK	S	925	E,D *
AT3G13530	M3KE1	mitogen-activated protein kinase kinase kinase 7	PM	Protein modification	S[+80]GQLDPNNPIFGQNETSSLSMIDQPDVLK	S	788	C,A *
AT5G19010	MPK16	mitogen-activated protein kinase 16	PM	Protein modification	VAFNDTPTAIFWTDY[+80]VATR	Y	189	D,A *
AT1G18150	MPK8	Protein kinase superfamily protein	PM	Protein modification	AAAAVASTLESEEADNGGGYS[+80]AR	S	539	D *
AT2G31010	Raf13	Protein kinase superfamily protein	PM	Protein modification	KLSNTSHS[+80]EPNVATVFWR	S	335	D *
AT1G16270	Raf18	kinase superfamily with octicosapeptide	PM	Protein modification	NT[+80]LVSGGVRGTLPWMAPELLNGSSSKVSEK	T	1024	E *,D *
AT2G24360	Raf22	Protein kinase superfamily protein	PM	Protein modification	HYS[+80]LSVGQSVFRPGR	S	81	A *
AT5G50000	Raf33	Protein kinase superfamily protein	PM	Protein modification	LLDWGEEGHRS[+80]EAEIVSLR	S	120	C *
AT5G58950	Raf36	Protein kinase superfamily protein	PM	Protein modification	SVS[+80]PSPQMAVPDVFK	S	101	C *
AT3G58760	Raf47	Integrin-linked protein kinase family	PM	Protein modification	SSGS[+80]FNR	S	468	A *
AT4G35310	CPK5	Calcium-dependent protein kinase 5	PM	Protein modification	NSLNIS[+80]MRDA	S	552	A *
AT1G49580	CRK8	Calcium-dependent protein kinase (CDPK) family protein	PM	Protein modification	TES[+80]GIFR	S	360	A *
AT4G04720	CPK21	calcium-dependent protein kinase 21	PM	Protein modification	T[+80]MFANIDTDK	T	387	A *
AT2G17290	CPK6	Calcium-dependent protein kinase family protein	C,PM	Protein modification	NSLNIS[+80]MRDV	S	540	E,D *
AT5G12480	CPK7	calmodulin-domain protein kinase 7	PM	Protein modification	FNSLS[+80]LKLMR	S	520	E,C *
AT3G10660	CPK2	calmodulin-domain protein kinase cdpk isoform 2	ER,EM	Protein modification	VSSAGLRT[+80]ESVLQRK	T	171	C *
AT3G19100	CRK2	Protein kinase superfamily protein	PM	Protein modification	DAVLQNDDSTPAHPGKS[+80]PVR	S	37	D *
AT5G58380	CIPK10	SOS3-interacting protein 1	C,PM	Protein modification	KS[+80]NGDTLEYQK	S	421	B *
AT3G09830	PCRK1	Protein kinase superfamily protein	PM	Protein modification	IVEASSGNGS[+80]PQLVPLNSVK	S	377	A *
AT2G01820	TMK3	Leucine-rich repeat protein kinase family protein	PM	Protein modification	LAPDGKYS[+80]IETR	S	745	A *
AT5G18610	PBL27	Protein kinase superfamily protein	PM	Protein modification	LGPVGDKTHVS[+80]TR	S	244	B *
AT1G66150	TMK1	transmembrane kinase 1	PM	Protein modification	EASFKKAIDT[+80]T[+80]IDLDEET[+80]LASVHTVAELAGHCCAR	T;T;T	825;826;833	C *
AT2G39660	BIK1	botrytis-induced kinase1	PM	Protein modification	LDTQYLPEEAVRMASVAVQCLS[+80]FEPKSRPTMDQVVR	S	333	C *
AT3G53380	LECRK81	Concanavalin A-like lectin protein kinase family protein	PM	Protein modification	QIEHDKS[+80]PEATVAAGTMGYLAPEYLLTGR	S	530	C *
AT4G23250	EMB1290	cysteine-rich receptor-like protein kinase 17	PM	Protein modification	IFGVDQTVANT[+80]AR	T	518	D *
AT2G37710	LECRK41	receptor lectin kinase	PM	Protein modification	LYDHGSDPQTT[+80]HVVGTLGYLAPEHTR	T	506	D *
AT5G16590	LRR1	Leucine-rich repeat protein kinase family protein	PM	Protein modification	WVSSITEQQS[+80]PSDVFDPELTR	S	562	E,B *
AT5G41210	GSTT1	glutathione S-transferase THETA 1	Per	Protein modification	REMGTLSKPGLQS[+80]KI	S	243	E,C *
AT1G72710	CKL2	casein kinase 1-like protein 2	PM	Protein modification	NSGQIFNS[+80]GSLAK	S	353	C *
AT4G26540	RGI3	Leucine-rich repeat receptor-like protein kinase family protein	PM	Phytohormone action	NLTS[+80]ANVIGTGSSGVVYR	S	762	B *
AT2G13790	SERK4	somatic embryogenesis receptor-like kinase 4	PM	Phytohormone action	LMNYNDS[+80]HVTTAVR	S	451	B *
AT5G38990	AT5G38990	Malectin/receptor-like protein kinase family protein	PM	Phytohormone action	VGPTSAS[+80]QTHVSTVVK	S	683	C *
AT5G59700	AT5G59700	Protein kinase superfamily protein	PM	Phytohormone action	ANPKSQQGLAEFRT[+80]EIEMLSQFR	T	525	D *
AT1G06390	ASK9	GSK3/SHAGGY-like protein kinase 1	PM	Phytohormone action	VLVKGEPNIS[+80]YICSR	S	229	B *
AT1G01740	BSK4	kinase with tetratricopeptide repeat domain-containing protein	PM	Phytohormone action	SYS[+80]TNLAFTPPEYLR	S	210	E,C *
AT4G18710	ASK7	Protein kinase superfamily protein	PM	Phytohormone action	QLVKGEANISY[+80]ICSR	Y	200	D *
AT1G70940	PIN3	Auxin efflux carrier family protein	PM	Phytohormone action	ELHMFVWSSNGS[+80]PVSDR	S	366	D *
AT2G01420	PIN4	Auxin efflux carrier family protein	PM	Phytohormone action	MVVSDQPRKS[+80]NAR	S	395	E,C *
AT2G01420	PIN4	Auxin efflux carrier family protein	PM	Phytohormone action	KS[+80]GGDDIGGLDSGEGEREIEK	S	395	C *
AT1G23080	PIN7	Auxin efflux carrier family protein	PM	Phytohormone action	LRCNS[+80]TAELNPK	S	431	E,C *
AT1G23080	PIN7	Auxin efflux carrier family protein	PM	Phytohormone action	SFYGGGGTNMTPRPSNLTGAEIYSLNTT[+80]PR	T	242	C *
AT4G33240	FAB1A	1-phosphatidylinositol-3-phosphate 5-kinase FAB1A	G,EM	Multi-process regulation	NVS[+80]LEKLSDEKVK	S	1143	C *
AT1G71010	FAB1C	FORMS APLOID AND BINUCLEATE CELLS 1C	C,EM	Multi-process regulation	VQS[+80]FDSAIR	S	1184	A *
AT1G49340	PI4KA1	Phosphatidylinositol 3- and 4-kinase family protein	PM	Multi-process regulation	LIS[+80]GAFSQAPQPEDDSFNEMLIAR	S	1111	D *
AT1G60890	AT1G60890	Phosphatidylinositol-4-phosphate 5-kinase family protein	PM	Multi-process regulation	AFS[+80]VGEKEVDLILPGTAR	S	662	C *
AT3G01310	AT3G01310	Phosphoglycerate mutase-like family protein	G,PM	Multi-process regulation	QGS[+80]GIIGTFGQSEELR	S	358	B *
AT1G22620	SAC1	Phosphoinositide phosphatase family protein	G,EM	Multi-process regulation	ASQLSHANTAREPS[+80]LRDLR	S	456	A *
AT2G18730	DGK3	diacylglycerol kinase 3	PM	Lipid metabolism	FVAS[+80]RPSTADSKTMR	S	18	A *
AT3G08510	PLC2	phospholipase C 2	PM	Lipid metabolism	RLS[+80]LSEEQLEK	S	346	D
AT5G60900	RLK1	receptor-like protein kinase 1	PM	Enzyme classification	GAFGIVYKGYLEVAGGSEVT[+80]VAVKK	T	559	D *
AT1G17580	XI-1	myosin 1	C,CT	Cytoskeleton organization	QQTLTIS[+80]PTTR	S	1052	E,D *
AT3G19960	ATM1	myosin 1	PM	Cytoskeleton organization	S[+80]LPADYRFDGSPVSDRLENSSGASVR	S	14	E,C *
AT5G20490	XI-K	myosin family protein with Dil	G,EM	Cytoskeleton organization	ENS[+80]GFGFLLTRK	S	1516	C *
AT1G08730	XI-C	Myosin family protein with Dil domain-containing protein	C,CT	Cytoskeleton organization	KLHVASLVVQT[+80]GLR	T	823	C *
AT1G24764	ATMAP70-2	microtubule-associated proteins 70-2	MT	Cytoskeleton organization	GTSKS[+80]FDGGTR	S	464	C *
AT4G27060	TOR1	ARM repeat superfamily protein	C,MT	Cytoskeleton organization	EASDGSTLS[+80]PDSASKGK	S	370	E,A *
AT2G01750	MAP70-3	microtubule-associated proteins 70-3	PM	Cytoskeleton organization	MS[+80]EKLKLTENLLDS[+80]K	S;S	125;137	E,C *
AT4G29810	ATMKK2	MAP kinase kinase 2	PM	Cell cycle organization	IISQLEPEVLS[+80]PIKPADDQLSLSDLDMVK	S	56	D *
AT5G55230	ATMAP65-1	microtubule-associated proteins 65-1	MT	Cell cycle organization	RLS[+80]LNANQNGSR	S	532	C *

Bin names are extracted from the MAPMAN4.0 functional classification of proteins. K-means Cluster E represents peptides phosphorylated during at least three time points of cold exposure; the cluster number next to E represents the clusters where the phosphopeptide intensity was highest. Asterisk (*) indicates statistical significance (*p* < 0.05) by Student’s *t*-test. Localizations—PM: plasma membrane; G: Golgi; ER: endoplasmic reticulum; V: vacuole; Per: peroxisome; CT: cytoskeleton; MT: microtubule; EM: endomembrane system; PD: plasmodesmata; C: cytosol. For more detailed information, see Table S1. Detailed explanations on the K-means cluster names are in Figure 2. TAIR: the Arabidopsis information resource.

## Supplementary Materials

Supplementary Materials can be found at https://www.mdpi.com/1422-0067/21/22/8631/s1.

## Abbreviations

MMF	Microsomal membrane fraction
LC-ESI-MS/MS	Liquid chromatography–electrospray ionization mass spectrometry/mass spectrometry
