# Supplementary material for: Large-Scale Phosphoproteomic Study of Arabidopsis Membrane Proteins Reveals Early Signaling Events in Response to Cold
Source: Int J Mol Sci. 2020 Nov 16;21(22):8631. doi: 10.3390/ijms21228631 (PMC7696906; doi:10.3390/ijms21228631)
Supplement: Supplementary file 1 [file ijms-21-08631-s001.zip › ijms-1001458 supl/20201030_IJMS_Kamal_Supplemetary_figures_and_tables_information.pdf]

International Journal of
Molecular Sciences

MDPI

Article

Large scale phosphoproteomic study of *Arabidopsis* membrane proteins reveals early signaling events in response to cold

Md Mostafa Kamal [1], Shinnosuke Ishikawa [2], Fuminori Takahashi [3], Ko Suzuki [4], Masaharu Kamo [4], Taishi Umezawa [2], Kazuo Shinozaki [3], Yukio Kawamura [1,5], and Matsuo Uemura [1,5,*]

[1] United Graduate School of Agricultural Sciences, Iwate University, Morioka 020-8550, Japan; mostafa.kamal42@gmail.com
[2] Graduate School of Bio-Applications and Systems Engineering, Tokyo University of Agriculture and Technology, Koganei 184-8588, Japan; shinnosuke-ishikawa@keio.jp (S.I); taishi@cc.tuat.ac.jp (T.U)
[3] Gene Discovery Research Group, RIKEN Center for Sustainable Resource Science, 3-1-1 Koyadai, Tsukuba 305-0074, Japan; fuminori.takahashi@riken.jp (F.T.); kazuo.shinozaki@riken.jp (K.S.)
[4] Department of Biochemistry, Iwate Medical University, Yahaba 028-3694, Japan; kosuzu@iwate-med.ac.jp (K.S.); mkamo@iwate-med.ac.jp (M.K.)
[5] Department of Plant-bioscience, Faculty of Agriculture, Iwate University, Morioka 020-8550, Japan; ykawa@iwate-u.ac.jp (Y.K.); uemura@iwate-u.ac.jp (M.U.)

* Correspondence: uemura@iwate-u.ac.jp; Tel.: +81-19-621-69253

Information on supplementary tables

Table S1. Identified cold-responsive phosphopeptides in the microsomal membrane fraction (MMF). (A) Class-I phosphopeptides based on Mascot variable localization confidence score ≥ 0.75. (B) All phosphopeptides identified in the study using Skyline-Daily software.

Table S2. Identified proteins from microsomal membrane fraction (MMF). Data were generated using Progenesis software for proteomics data analysis. The data show no significant (anova P value ≤ 0.05) changes in protein abundance within a short duration of cold exposure.

Table S3. An updated *Arabidopsis* kinase-substrate interaction network from published literature. (A) Binary interaction between kinase and corresponding substrates was used to construct the kinase-substrate network. (B) Sub-network data were used to build the cold-responsive kinase-substrate network shown in Figure 6. (C) The number of binary interactions between a kinase and its corresponding substrates.

International Journal of
Molecular Sciences

MDPI

Information on supplementary figures

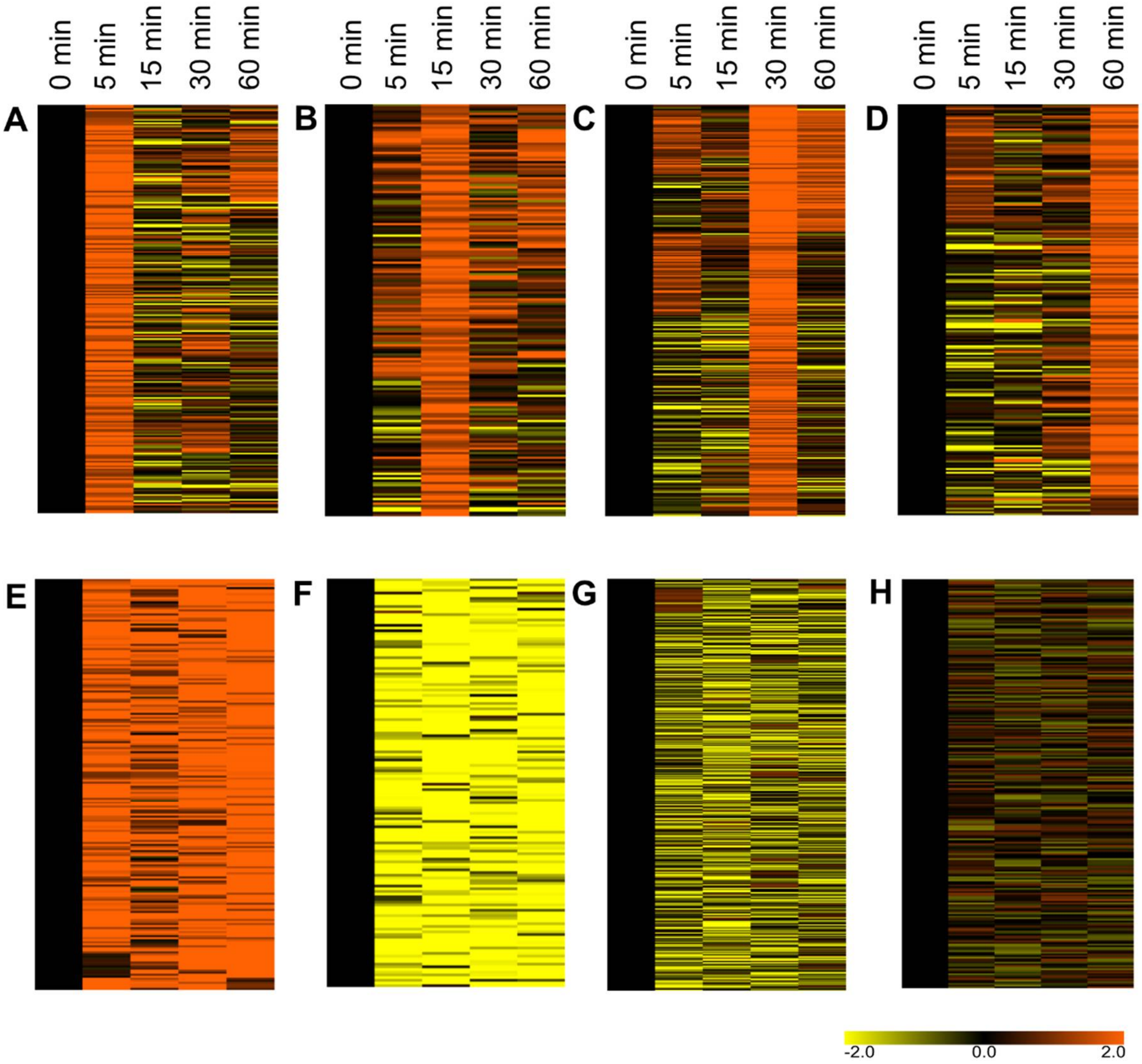

Figure S1. K-means clusters of differentially expressed phosphopeptides (DEPS) in response to cold at different time points. Supplementary to Figure 2. Times at the top indicate the duration of cold exposures. The scale indicates the log2 fold change in the cold exposed groups mentioned in Figure 2. (A) Fold change increased at 5 min. (B) Fold change increased at 15 min. (C) Fold change increased at 30 min. (D) Fold change increased at 60 min (E) Continuous increase in fold change under cold exposure; (F) Continuous decrease in fold change under cold exposure. (G) Fold change decreased under cold exposure, but not continuously. (H) Unresponsive to cold exposure.

International Journal of
Molecular Sciences

MDPI

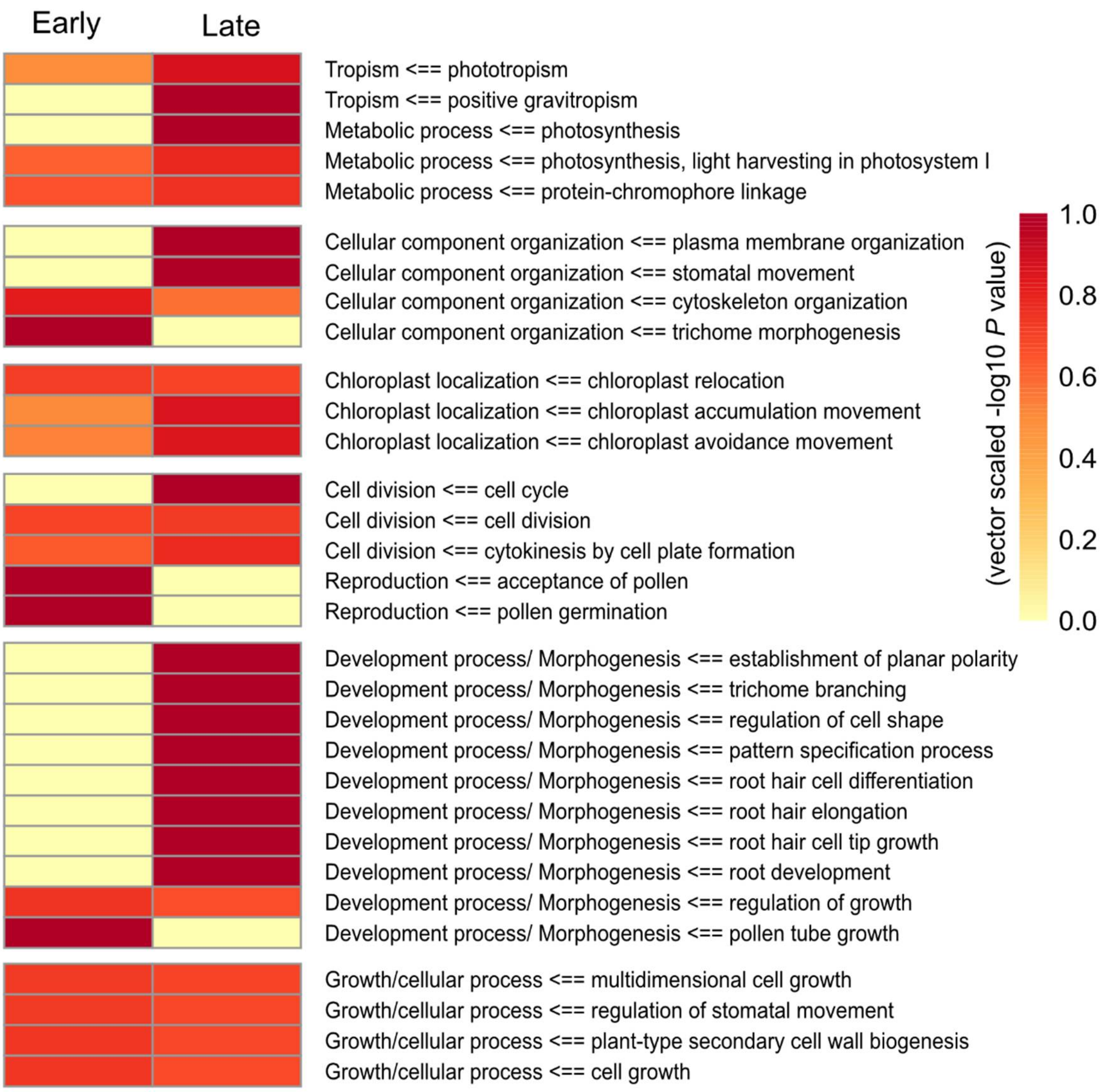

Figure S2. Gene ontology over-representation analysis of biological processes (GOBP). Continuation of Figure 4. Negative log 10 of *P* -values of GO terms enriched in the K-means clusters mentioned in Figure 4 were vector scaled and visualized. GO terms enriched within 5 to 15 min of cold exposure; Late, GO terms enriched within 30 to 60 min of cold exposure.

International Journal of
Molecular Sciences

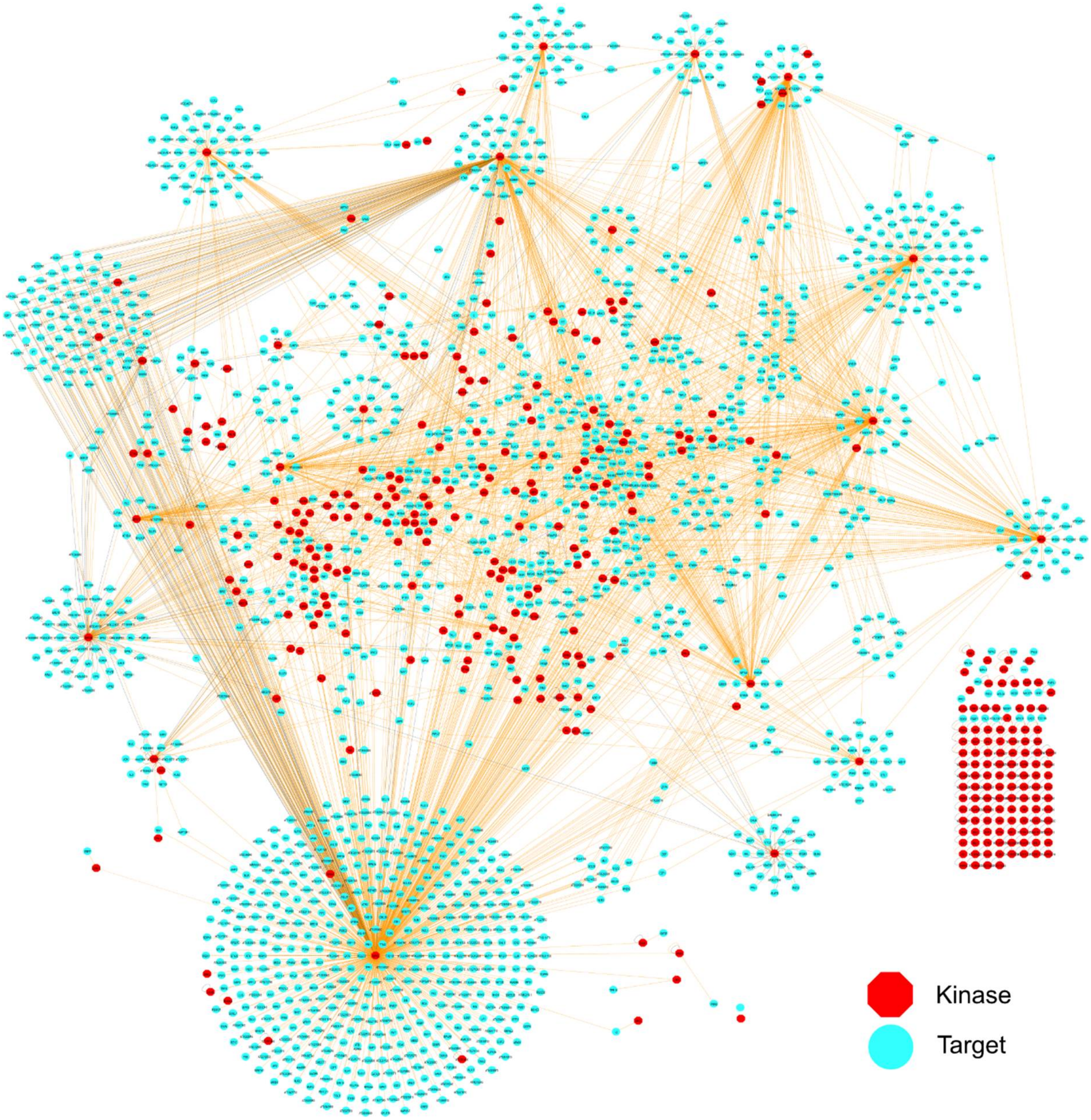

Figure S3. *Arabidopsis* kinase-substrate interaction network. This was constructed using available information from the PhosphAT database and other literature cited in the text. The subnetwork of *Arabidopsis* cold-responsive kinase-substrate interaction in Figure 6 was extracted based on this network.

International Journal of
Molecular Sciences

MDPI

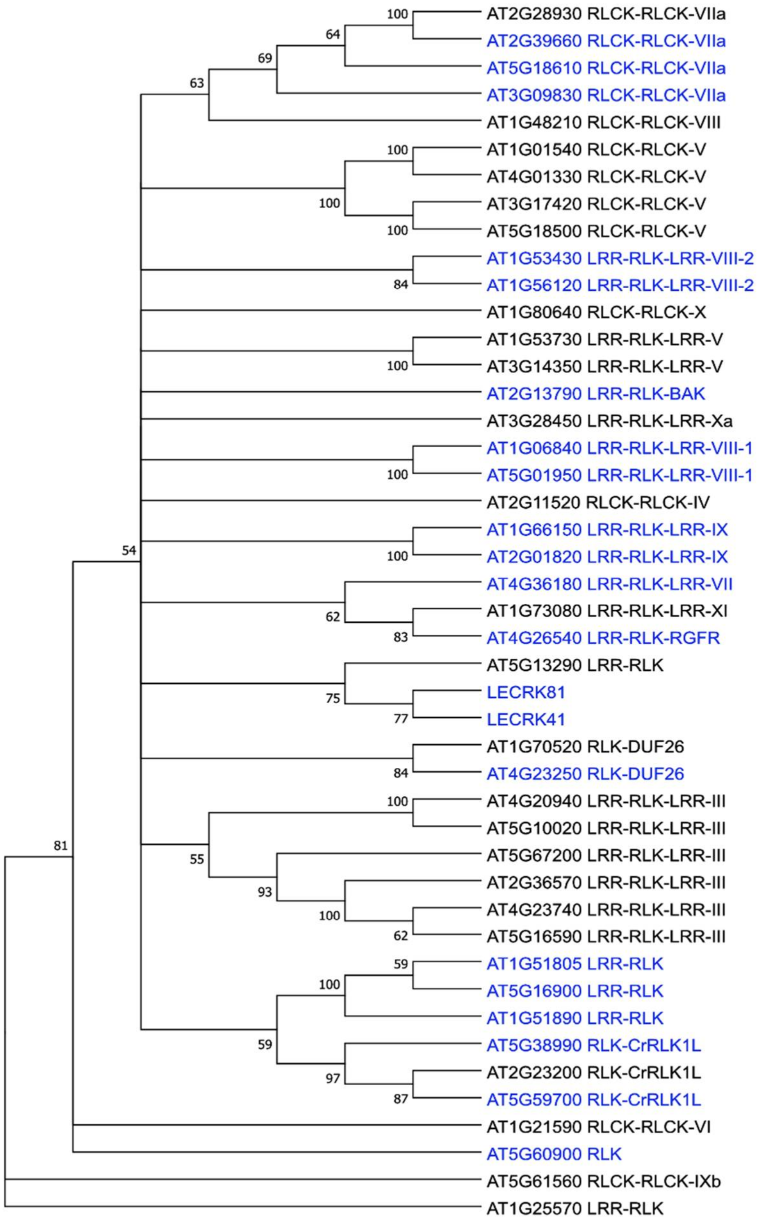

Figure S4. Coordinated regulation of related receptor kinase (RKs) gene family members (RLKs, RLCKs) in response to cold. The RLK/RLCKs phosphorylated inside their kinase domains are highlighted in blue. The closely related kinases may work in coordination under cold. The tree was made from protein sequence alignment using MEGA10 software.

International Journal of
Molecular Sciences

MDPI

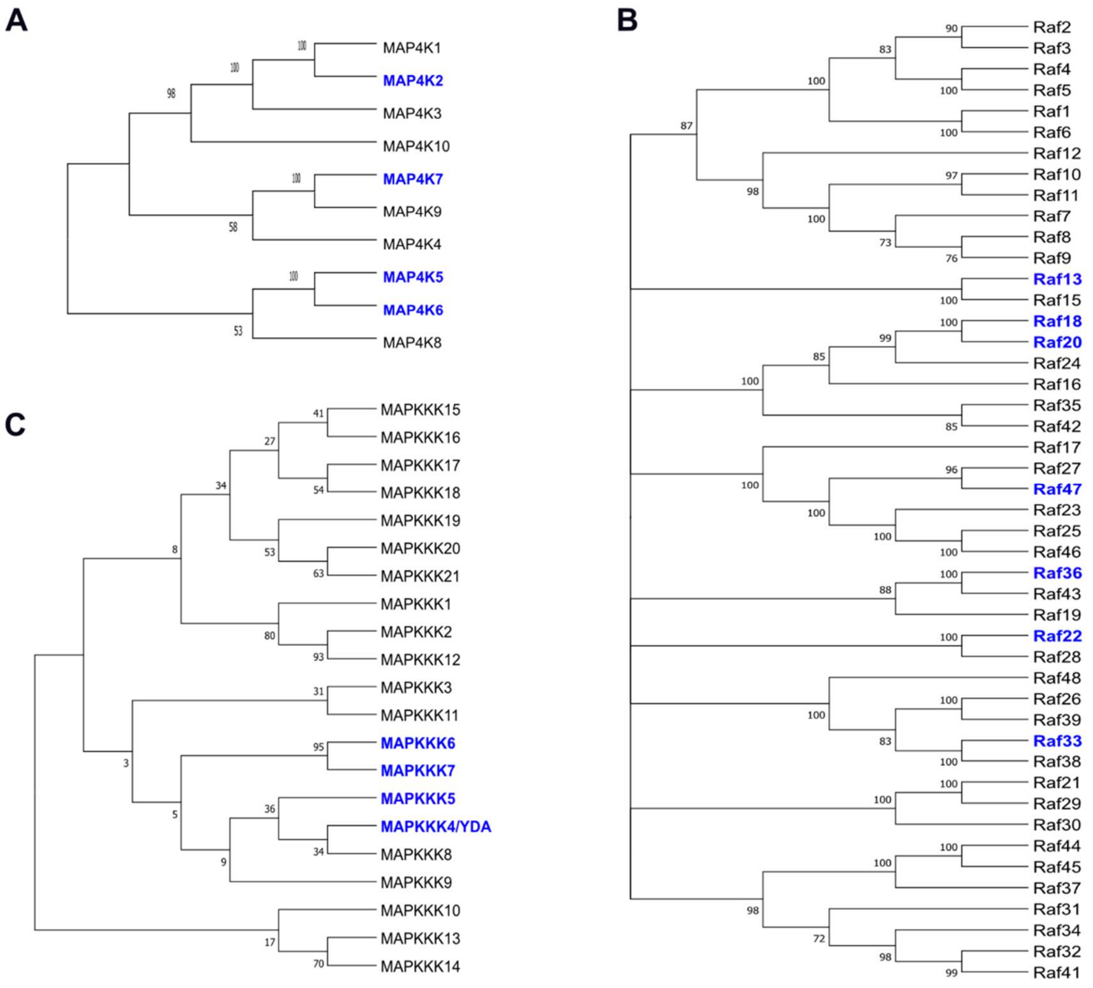

Figure S5. Coordinated regulation of related MAPK gene family members in response to cold. (A) MAP4K phylogenetic tree. (B-C) MAP3K and MAP3K RAF kinase phylogenetic tree. Kinases identified in this study are highlighted in blue. The closely related kinases may work in coordination under cold. The tree was made from protein sequence alignment using MEGA10 software.
